# The telomere maintenance mechanism spectrum and its dynamics in gliomas

**DOI:** 10.1186/s13073-022-01095-x

**Published:** 2022-08-11

**Authors:** Sojin Kim, Tamrin Chowdhury, Hyeon Jong Yu, Jee Ye Kahng, Chae Eun Lee, Seung Ah. Choi, Kyung-Min Kim, Ho Kang, Joo Ho Lee, Soon-Tae Lee, Jae-Kyung Won, Kyung Hyun Kim, Min-Sung Kim, Ji Yeoun Lee, Jin Wook Kim, Yong-Hwy Kim, Tae Min Kim, Seung Hong Choi, Ji Hoon Phi, Young-Kyoung Shin, Ja-Lok Ku, Sungyoung Lee, Hongseok Yun, Hwajin Lee, Dokyoung Kim, Kyoungmi Kim, Junho K. Hur, Sung-Hye Park, Seung-Ki Kim, Chul-Kee Park

**Affiliations:** 1 Department of Neurosurgery, Seoul National University College of Medicine, Seoul National University Hospital, 101 Daehak-ro, Jongno-gu, Seoul, 03080 Republic of Korea; 2 Seoul National University College of Medicine, Seoul, 03080 Republic of Korea; 3 Division of Pediatric Neurosurgery, Pediatric Clinical Neuroscience Center, Seoul National University Children’s Hospital, Seoul, 03080 Republic of Korea; 4 Department of Radiation Oncology, Seoul National University Hospital, Seoul, 03080 Republic of Korea; 5 Department of Neurology, Seoul National University Hospital, Seoul, 03080 Republic of Korea; 6 Department of Pathology, Seoul National University Hospital, Seoul, 03080 Republic of Korea; 7 Department of Anatomy and Cell Biology, Seoul National University College of Medicine, Seoul, 03080 Republic of Korea; 8 Department of Internal Medicine, Seoul National University Hospital, Seoul, 03080 Republic of Korea; 9 Department of Radiology, Seoul National University Hospital, Seoul, 03080 Republic of Korea; 10 Korean Cell Line Bank, Laboratory of Cell Biology, Cancer Research Institute, Seoul National University College of Medicine, Seoul, 03080 Republic of Korea; 11 Department of Genomic Medicine, Seoul National University Hospital, Seoul, 03080 Republic of Korea; 12 Biomedical Knowledge Engineering Laboratory and Dental Research Institute, Seoul National University, Seoul, 08826 Republic of Korea; 13 Department of Anatomy and Neurobiology, College of Medicine, Kyung Hee University, Seoul, 02447 Republic of Korea; 14 Department of Biomedical Sciences and Department of Physiology, Korea University College of Medicine, Seoul, 02841 Republic of Korea; 15 Department of Genetics, College of Medicine, Hanyang University, Seoul, 04763 Korea; 16 Genomic Medicine Institute, Medical Research Center, Seoul National University, Seoul, Korea

**Keywords:** Glioma, Telomere maintenance mechanism, TERT, ALT

## Abstract

**Background:**

The activation of the telomere maintenance mechanism (TMM) is one of the critical drivers of cancer cell immortality. In gliomas, *TERT* expression and *TERT* promoter mutation are considered to reliably indicate telomerase activation, while *ATRX* mutation and/or loss indicates an alternative lengthening of telomeres (ALT). However, these relationships have not been extensively validated in tumor tissues.

**Methods:**

Telomerase repeated amplification protocol (TRAP) and C-circle assays were used to profile and characterize the TMM cross-sectionally (*n* = 412) and temporally (*n* = 133) across glioma samples. WES, RNA-seq, and NanoString analyses were performed to identify and validate the genetic characteristics of the TMM groups.

**Results:**

We show through the direct measurement of telomerase activity and ALT in a large set of glioma samples that the TMM in glioma cannot be defined solely by the combination of telomerase activity and ALT, regardless of *TERT* expression, *TERT* promoter mutation, and *ATRX* loss. Moreover, we observed that a considerable proportion of gliomas lacked both telomerase activity and ALT. This telomerase activation-negative and ALT negative group exhibited evidence of slow growth potential. By analyzing a set of longitudinal samples from a separate cohort of glioma patients, we discovered that the TMM is not fixed and can change with glioma progression.

**Conclusions:**

This study suggests that the TMM is dynamic and reflects the plasticity and oncogenicity of tumor cells. Direct measurement of telomerase enzyme activity and evidence of ALT should be considered when defining TMM. An accurate understanding of the TMM in glioma is expected to provide important information for establishing cancer management strategies.

**Supplementary Information:**

The online version contains supplementary material available at 10.1186/s13073-022-01095-x.

## Background

The immortal nature of cancer cells relies on the maintenance of telomere length to allow unlimited replication, preventing cellular senescence [1, 2]. There are two recognized telomere maintenance mechanisms (TMMs) in cancer: telomerase activation, which is known to exist in 85–90% of malignancies [3, 4], and a homologous recombination-based process called alternative lengthening of telomeres (ALT) [5]. The genetic and epigenetic mechanisms of telomerase activation in cancer include somatic telomerase reverse transcriptase (*TERT*) alterations, such as *TERT* promoter (*TERTp*) mutation, *TERT* amplification, *TERT* or *TERTp* structural variations, and *TERTp* hypermethylation, which induce the expression of *TERT* [6]. Among them, *TERTp* mutation is considered the major mechanism of telomerase activation in glioblastoma (GBM) and lower-grade glioma (LGG) [4, 6–8]. Moreover, GBM and oligodendroglioma are among the cancers with the highest frequencies (60–80%) of *TERTp* mutations [9–11]. However, approximately 15% of GBMs use ALT as the TMM, and pediatric gliomas show a higher frequency of ALT [5, 12]. It is widely believed that ALT in cancers is tightly connected with inactivating mutations in the *ATRX* gene as shown by previous studies [13, 14] and that the presence of *TERTp* mutation and *ATRX* mutation are mutually exclusive in gliomas [15].

Growing evidence shows that some gliomas harbor neither *TERTp* nor *ATRX* mutations [15]. Similar findings were observed in other cancers. A systematic analysis of the whole-genome data of 31 cancer types revealed that 22% of cancers have neither *TERT* expression nor *ATRX* mutations (8.1% of GBMs and 10.2% of LGGs) [6]. The functional TMM in this subgroup of cancers has not been well studied. On the other hand, a minority of cancer cases, including GBMs, have both telomerase activation and ALT as TMMs [16]. In addition, to date, many results have been reported under the assumption that telomerase enzyme activity is equivalent to *TERT* expression or *TERTp* mutation in gliomas, but this paradigm has been challenged recently [17]. Similarly, the loss of *ATRX* expression alone is not sufficient to trigger ALT, implying that *ATRX* mutation is not both necessary and sufficient for ALT [18].

There is increasing evidence that the TMM in cancer cells may not be a fixed feature. Many in vitro and in vivo studies have shown the phenomenon of TMM switching between telomerase activation and ALT [19–25]. TMM switching can be induced by anticancer treatment [21] or can accompany the transdifferentiation of cancer cells, such as epithelial-mesenchymal transition (EMT) [19]. Although there is evidence of spatiotemporal consistency in *TERTp* and *ATRX* mutations in gliomas [26, 27], changes in the TMM have not been thoroughly documented using longitudinal samples of human cancer. Moreover, the pressures that drive the switching of the TMM during cancer development or treatment are unclear, and the biological effects of the switching of the TMM and its impact on cancer management or prognosis are still unknown.

To acquire this fundamental knowledge and characterize the landscape of the TMM in gliomas, we profiled the TMM status of the whole spectrum of gliomas in samples collected from patients of all ages at initial diagnosis. TMM was identified by the telomerase repeated amplification protocol (TRAP) for telomerase activity and the C-circle assay (CCA) for ALT activity in glioma tissues, and their associations with genetic characteristics and clinical variables were investigated. We confirmed that TMM status does not always correlate with signatures such as *TERTp* mutation or *ATRX* loss in gliomas. Telomerase activation and ALT are not mutually exclusive in gliomas, and some gliomas employ neither or both of these TMMs. We uncovered TMM characteristics related to age and identified their prognostic power in gliomas. In cases when neither telomerase nor ALT was activated, we also explored the genetic signatures that contribute to the maintenance of telomere length by genome sequencing analysis and characterized their biological behavior. Using another cohort composed of longitudinal paired samples of gliomas, we confirmed that TMM switching occurred during the progression of glioma. Collectively, the TMM is not an immutable feature governed by a specific genetic signature but rather has a dynamic nature with a variety of spectra according to the state of glioma cells.

## Methods

### Sample collection and diagnosis

A total of 476 glioma patient samples (412 newly diagnosed and 64 paired longitudinal) treated in Seoul National University Hospital were used in this study. Fresh tumor tissues were obtained from patients undergoing surgical resection with informed consent for their usage for research purposes in accordance with the guidelines of the Institutional Review Board of Seoul National University Hospital, which approved this study (IRB Nos. H-0507-509-153 and H-1608-139-787). Tissues were fresh frozen in liquid nitrogen immediately after resection, and white blood cells (WBCs) were extracted from the whole blood. Both tissue and WBC samples were then stored at − 80 °C for later use. The histological diagnoses of all gliomas were made according to the WHO 2016 classification. Each tissue sample was collected from the tumor core area differentiated by 5-aminolevulinic acid fluorescence intraoperatively and histologically confirmed that it was a tumor core sample rich in cancer cells after resection.

General genetic characterization of the cases was performed on all samples according to a routine diagnostic process, and the information was obtained from the medical record. The diagnostic tests included 1p/19q fluorescence in situ hybridization (FISH) to detect chromosome 1p/19q codeletion; IDH1/IDH2 direct sequencing (only when the IDH1 immunohistochemistry result was negative); FISH to detect CDKN2A (9p21) gene deletion, epidermal growth factor receptor (EGFR) (7p12) gene amplification, and PTEN (10q23) gene deletion; O6-methylguanine DNA methyltransferase (MGMT) methylation-specific polymerase chain reaction (PCR) analysis; and immunohistochemistry using primary antibody against MIB-1 (Ki-67) (Dako, 1:1000) to determine the proliferation index. All these molecular testing methods have been described previously [28]. Additionally, 24 individual GBM samples were randomly selected for RNA-seq according to their TMM status and 7 pairs of longitudinal ODG samples were selected for WES and RNA-seq for further analysis.

### DNA and RNA extraction and quantification

DNA was extracted from frozen tumor tissues and WBC samples with a Qiagen QIAamp DNA Mini Kit (Qiagen, Valencia, CA), and total RNA was extracted using an RNeasy Plus Mini Kit (Qiagen) and an RNeasy Lipid Tissue Mini Kit (Qiagen). DNA and RNA yield and purity were assessed using a DS 11 Spectrophotometer (Denovix Inc., DE, USA). The extracted DNA and RNA were then used in subsequent assays and sequencing experiments. The amount of total RNA recommended for use in NanoString was > 100 ng, as this quantity of input material generates a robust signal for most tissue/cell isolates. One hundred nanograms of total RNA was added to the sample preparation reaction in a 5-μL volume, and RNA quality was assessed using a fragment analyzer (Advanced Analytical Technologies, IA, USA).

### *TERTp* mutation

*TERTp* mutation analysis was performed as previously described [29]. Genomic DNA (gDNA) was extracted from 412 specimens using the QIAamp DNA Mini Kit (Qiagen), and purified gDNA was amplified using primers [5′-CTGGCGTCCCTGCACCCTGG-3′ (forward) and 5′-ACGAACGTGGCCAGCGGCAG-3′ (reverse)] and AccuPower® PCR PreMix (Bioneer). The thermal cycling conditions were as follows: 96 °C for 5 min, 35 cycles of 95 °C for 40 s, 68 °C for 30 s, and 72 °C for 30 min, followed by 72 °C for 5 min and a 4 °C infinite hold. PCR products were gel-purified, and Sanger sequencing was performed to screen for two *TERTp* hotspot mutations (C228T and C250T).

### *ATRX* loss

Immunohistochemistry was performed to detect *ATRX* loss using antibodies against ATRX (rabbit polyclonal, 1:600; Sigma-Aldrich, St. Louis, MO, USA). Sections from known mutation-positive and immunoreactive GBM tumors were used as positive controls. Sections incubated with normal rabbit serum instead of the primary antibody were used as negative controls. ATRX was scored as negative if staining loss was observed in > 90% of tumor cells. Immunoreactivity was assessed using the Aperio ImageScope software with the Nuclear v9 algorithm (Aperio Technologies, Vista, CA, USA).

### Telomere repeat amplification protocol (TRAP) assay with ELISA

Telomerase enzymatic activity was measured using a *TeloTAGGG* Telomerase PCR ELISA PLUS Kit (Roche) according to the manufacturer’s protocol. Glioma tissues and cells were homogenized in an ice-cold lysis buffer using an automill (Tokken). Briefly, after BCA protein quantification of the lysates, 10 μg of protein was incubated in a reaction mixture (total volume 50 μl) at 25 °C for 30 min to allow telomerase to add telomeric repeats to the end of the biotin-labeled primer. Subsequently, PCR was conducted for 33 cycles of 94 °C for 30 s, 50 °C for 30 s, and 72 °C for 90 s, followed by an additional extension time of 10 min at 72 °C and hold at 4 °C. Telomerase activity was measured at 450 nm and a reference wavelength of 690 nm. The relative telomerase activity (RTA) of each sample was calculated according to the instructions provided with the TeloTAGGG Telomerase PCR ELISA PLUS Kit. RTA% was calculated with the following formula: RTA% = (TA-heated TA/internal CTR)/(CTR-lysis buffer/internal CTR) × 100. We considered a sample to be telomerase positive if the TA/heated TA ≥ 2. The RTA calculation was mainly a relative comparison of the telomerase activity of the samples compared to the internal standards. When a sample showed twice as much activity (RTA = 2) as the negative controls (telomerase deactivated), it was determined to be telomerase active.

### C-circle assay (CCA)

The detection of C-circles was performed as previously described [30]. Briefly, 30 ng of DNA was combined with 10 μl of 2X Φ29 buffer, 7.5 U of Φ29 DNA polymerase (NEB), 0.2 mg/ml BSA, 0.1% (v/v) Tween 20, and 1 mM each dATP, dGTP, and dTTP and incubated at 30 °C for 4 h or 8 h followed by 20 min at 70 °C. Amplification products were deposited onto a Hybond N+ nylon membrane (Bio-Rad) and developed using the TeloTAGGG Telomere Length Assay Kit (Roche). Chemiluminescent signals were visualized with a ChemiDoc XRS system (Bio-Rad), and the intensity of the spots was quantified with the ImageQuant TL software (Bio-Rad). The 293T and U2OS cell lines were used as negative and positive controls. The intensity of the spots was quantified with ImageJ Fiji (version 1.53c) [31]. The positive control spot intensity was fixed at 100, and all the other samples were calculated in comparison with the positive control. A sample was considered to be ALT-positive if it had a relative value of > 30, which corresponds to a clearly distinguishable intensity (Additional file 1: Fig. S1c).

### Next-generation sequencing and data analysis

DNA and mRNA qualities were checked with a Bioanalyzer (Agilent, CA, USA) and considered to be acceptable if the quantity ≥ ≥ 1 μg. For mRNA, RIN ≥ ≥ 7 and rRNA ratio ≥ 1 were also included as quality control criteria. After a quality control assessment of the samples, a sequencing library was prepared by random fragmentation of the DNA or cDNA, followed by 5′ and 3′ adapter ligation. Library preparation was performed using the SureSelectXT Library Prep Kit for whole-exome sequencing (WES) and the TruSeq Standard mRNA LT Sample Prep Kit for RNA-seq. All next-generation sequencing assays were performed using the Illumina platform at Macrogen (Seoul, Korea). The generated BCL binary was then converted into raw FASTQ files utilizing the Illumina bcl2fastq package. WES FASTQ files were mapped to the reference genome (UCSC hg19) with BWA (0.7.12), and PCR duplicates were marked with Picard (1.130) [7, 32]. Base recalibration and SNP and INDEL calling were performed using BQSR and Mutect2 in GATK (v4.1.9.0) [33]. To track clonal evolution across the 7 paired samples of the initial and progression of disease, the generated bam and vcf files were then analyzed with SuperFreq [34].

RNA-seq FASTQ files were quality checked and trimmed to remove adapters and low-quality reads using FastQC and Trimmomatic [35, 36]. The trimmed FASTQ files were mapped to the UCSC hg19 reference genome with HiSat2, and raw gene counts were calculated with StringTie [37, 38]. RNA-seq expression count data were normalized, and batch effects were removed with the edgeR and limma packages in R (version 3.5.1; http://www.r-project.org/) [39, 40]. Twenty-four TMM samples were clustered hierarchically using the ComplexHeatmap package of R [41]. Differential gene expression analysis was performed between 3 groups (telomerase, ALT, and negative) using the trend method of the limma package [42]. The log2-fold changes were calculated for each pair of the 7 paired switching samples by subtracting the log2 normalized expression values of the initial sample from that of the progressed sample. GSEA preranked analysis was performed with the 24 TMM samples ranking the genes according to the differential gene expression analysis [43, 44]. Canonical pathways and Gene Ontology gene sets from the Molecular Signatures Database (MSigDB) were used for GSEA. Both positively and negatively enriched GSEA results were first filtered according to the following criteria: FDR (< 0.0005) and *P* value (< 0.05). Then, the top 20 gene sets were selected from each group (negative vs. telomerase and negative vs. ALT) and correlated with each other to identify the significantly altered gene sets in the negative group.

### NanoString nCounter system mRNA expression profiling and analysis

The digital multiplexed NanoString nCounter human mRNA expression assay (NanoString Technologies) was performed with 100 ng of total RNA isolated from GBM tissues, cell lines, and the Custom code set. Hybridization was carried out by combining 5 μl of each RNA sample with 8 μl of nCounter Reporter probes in hybridization buffer and 2 μl of nCounter Capture probes (for a total reaction volume of 15 μl) overnight at 65 °C for 18 h. Excess probes were removed using two-step magnetic bead-based purification on the nCounter Prep Station (NanoString Technologies). The abundances of specific target molecules were quantified on the nCounter Digital Analyzer by counting the individual fluorescent barcodes and assessing the target molecules. For each assay, a high-density scan encompassing 280 fields of view was performed. The data were collected using the nCounter Digital Analyzer after taking images of the immobilized fluorescent reporters in the sample cartridge with a CCD camera. mRNA data normalization and analysis were performed using the nSolver (version 4.0) software, which is freely available from NanoString Technologies. The mRNA profiling data were normalized using housekeeping genes (*ACTB*, *GAPDH*, *GUSB*). The R software was used for the analysis.

### Analysis of TMM-related genes

TMM-related genes were downloaded from the Telnet database (http://www.cancertelsys.org/telnet/) [45]. We then extracted the normalized gene expression of the genes in the Telnet database among the sequenced samples (24 GBM samples with various TMMs). Among the 2090 genes in the Telnet database, 1940 genes were found in our sample; the other genes were filtered out due to low expression or poor sequencing quality among all samples during the normalization step. Further filtering was performed based on the differential gene expression analysis (LFC ≥ 1.5 or ≤ − 1.5 in the negative vs. telomerase and negative vs. ALT comparisons), and 53 significantly altered genes in the negative group were selected for the initial analysis. With the normalized expression values of the 53 TMM-related genes, we then carried out the principal component analysis (PCA) and plotted the results in R to identify TMM-related sample clusters.

### Cell lines

Nine patient-derived GBM cell lines (SNU-3980, 4026, 4054, 4254, 4317, 4327, 4638, 4509, and 4982) were established using a protocol previously described by the Korean Cell Line Bank (Seoul, Korea) [46]. The human osteosarcoma cell line U2OS was purchased from the Korean Cell Line Bank, and the human embryonic kidney cell line 293T was purchased from the American Type Culture Collection (ATCC). These cell lines were used as controls for the TRAP assay and C-circle assay. Cells were maintained in DMEM (Welgene) or RPMI-1640 medium (Gibco) supplemented with 10% fetal bovine serum (Gibco), 100 U/ml penicillin and 100 μg/ml streptomycin sulfate (Gibco).

### Cell proliferation assay

Cell proliferation assays were performed with EZ-Cytox (Daeillab Service) on cells initially plated at 1 × 10^3^ cells/well in 96-well plates and cultured as described above for the indicated times. The absorbance at a wavelength of 450 nm was measured using a microplate reader (Molecular Devices). Cell absorbance was measured at 2-day intervals for 21 days, and then the doubling time (DT) was calculated in R according to the following formula: DT = log(2)/linear regression slope.

### Telomere length estimation

Telomere length was determined by Southern blotting (TRF analysis) using a TeloTAGGG Telomere Length Assay Kit (Roche) according to the manufacturer’s protocol. Briefly, 1 μg of each DNA sample was digested with Rsa I and Hinf I overnight at 37 °C, electrophoresed on a 0.8% agarose gel at 50 V for 4 h and then transferred to a nylon membrane by Southern blotting. The blotting membrane was blocked and *hybridized overnight* to a digoxigenin (*DIG*)-labeled *probe* specific for telomeric repeats. The washed blot was incubated with anti-DIG-alkaline phosphatase (1:1000 dilution) for 30 min and developed using the substrate in the TeloTAGGG Telomere Length Assay Kit (Roche). The chemiluminescence signals were visualized with a ChemiDoc XRS system (Bio-Rad), and TRF analysis was performed with TeloTool version 1.3 [47].

### Statistics

Descriptive data statistics were visualized using the Tableau Desktop platform (version 2020.1, Tableau Software, Inc., Seattle, WA, USA). Comparative data analysis was conducted using one-way ANOVA with the Bonferroni correction *T* test. Survival analysis was performed, and cumulative survival statistics were calculated using Kaplan–Meier curves and log-rank tests. The significance level was set to *P* < 0.05, and all statistical analyses were performed in R (version 3.5.1; http://www.r-project.org/). We measured linear dependencies using the Pearson correlation coefficient as a metric for the correlation estimate, and we calculated the *P* value associated with each correlation. We derived the correlograms using the corrplot package in R. The optimal age and RTA cutoffs were assessed by the means of maximally selected log-rank statistics using the Maxstat package in R.

## Results

### The landscape of TMM profiles in gliomas

**Fig. 1:**
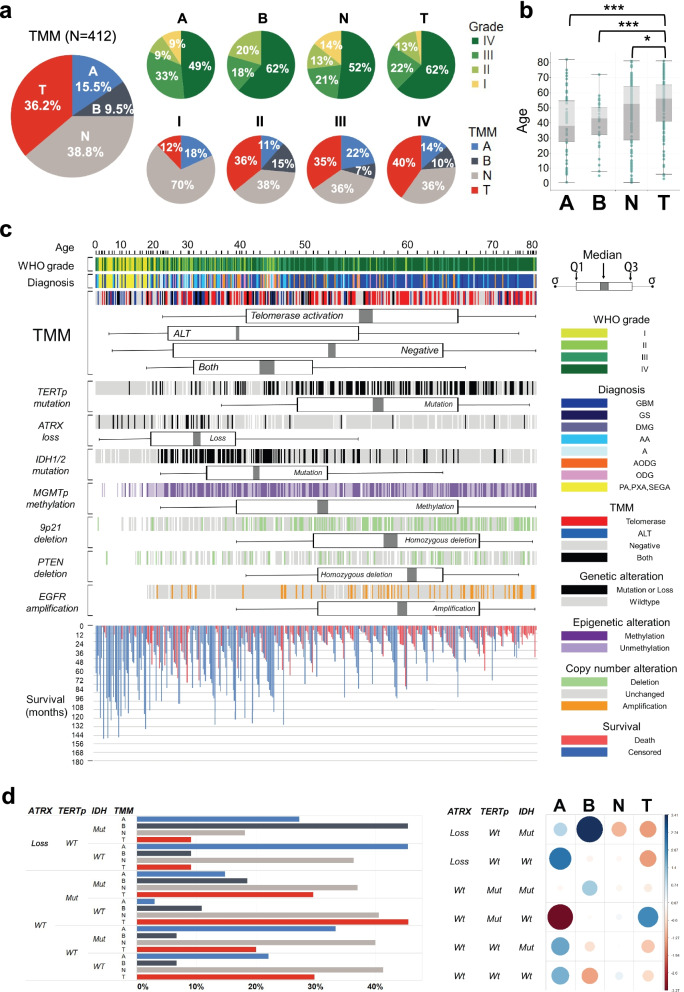
Clinical and genetic characteristics of telomere maintenance mechanism (TMM) groups in gliomas. T, telomerase activation; A, alternative lengthening of telomeres (ALT); B, both; N, negative. **a** The frequency of TMM groups among 412 glioma patients of all ages. The subdistribution of World Health Organization (WHO) classification grades (I~IV) of glioma among TMMs and vice versa are shown. **b** The age distribution of glioma patients in the TMM groups (ANOVA with Bonferroni correction *T* test; *p < 0.05, ***p < 0.001). **c** Landscape of TMM profiles and selected genetic and clinical characteristics and survival duration in all glioma patients, ordered by patient age. Boxplots indicate the median value, interquartile range, and standard deviation of the age distribution in a given condition. GBM, glioblastoma; GS, gliosarcoma; DMG, diffuse midline glioma; AA, anaplastic astrocytoma; A, astrocytoma; AODG, anaplastic oligodendroglioma; ODG, oligodendroglioma; PA, pilocytic astrocytoma; PXA, pleomorphic xanthoastrocytoma; SEGA, subependymal giant cell astrocytoma. **d** The distribution of major genetic signatures in gliomas among TMM groups and the associated correlogram. The color intensity and the size of the circle are proportional to the correlation coefficients and the amount of the cell contribution, respectively. Positive correlations are shown in blue, while negative correlations are shown in red (Pearson residuals, chi-squared = 49.026, df = 15, *p* < 0.001)

To gain insights into the TMM status across gliomas, we built a retrospective cohort comprising 412 newly diagnosed glioma patients of all ages (from 6 months to 82 years; Additional file 1: Fig. S1a) and various histological diagnoses (Additional file 1: Fig. S1b) from Seoul National University Hospital and secured fresh frozen tumor samples for TMM assessment. Based on the combined results of the CCA for ALT activity and the TRAP for telomerase activity, the samples were classified into 4 groups with different TMM statuses (Additional file 1: Fig. S1c): telomerase (*n* = 149, 36.2%), ALT (*n* = 64, 15.5%), negative (*n* = 160, 38.8%), and both (*n* = 39, 9.5%) (Fig. 1a). The telomerase group included patients with evident telomerase activity (relative telomerase activity (RTA) ≥ 2) and without any ALT activity (negative CCA), while the ALT group included patients without evident telomerase activity (RTA < 2) and with ALT activity (positive CCA). The both group included patients with both evident telomerase activity and ALT (RTA ≥ 2 and positive CCA), while the negative group included patients with limited evidence of telomerase and ALT activity (RTA < 2 and negative CCA). In terms of the interrelation between the TMM and the glioma grade based on the World Health Organization (WHO) classification [48], it is notable that all the TMM groups exhibited similar tumor grade distributions, except that the both group lacked patients with grade I tumors (Fig. 1a). We also analyzed the histological diagnoses of gliomas among the TMM groups and found that the negative group included nearly all glioma histology (Additional file 1: Fig. S1d and S1e).

We also noted that patients in the telomerase group were significantly older than those in the other TMM groups (Fig. 1b). To investigate this point further, we arranged the patients in order by age, and we found that the characteristics of the TMM and other related genetic factors clustered intuitively (Fig. 1c). Specifically, there was a clear pattern of predominant TMM type changing sequentially with patient age from the ALT group (with the youngest median age) to the telomerase group (Fig. 1c). However, the negative group had no specific age preponderance and was distributed across all ages, while the both group was mainly distributed among the young- and middle-aged patients (Fig 1c).

Unlike the signatures of *TERTp* mutation and *ATRX* loss, which are mutually exclusive in gliomas, and contrary to our expectation, the TMM groups could not be predicted by *TERTp* mutation or *ATRX* loss (Fig. 1c and Additional file 1: Fig. S2). However, correspondence analysis showed that the telomerase group consisted predominantly of *wtATRX/mutTERTp/wtIDH* gliomas, while the ALT group consisted predominantly of *ATRXloss/wtTERTp/wtIDH*, *wtATRX/wtTERTp/mutIDH*, and *wtATRX/wtTERTp/wtIDH* gliomas (Fig. 1d). Nonetheless, correspondence analysis results showed no significant enrichment of the negative group for *ATRX* loss, *TERTp* mutation, *IDH* mutation, or their combination. The individual relationships between the TMM and the mutational statuses of *TERTp*. *IDH* and *ATRX* loss are presented in Additional file 1: Fig. S3. In addition, the major genetic signatures mainly seen in gliomas such as *MGMT* promoter methylation, chromosome 9p21 deletion, *PTEN* deletion, and *EGFR* amplification did not show any special association with TMM. From the landscape view of the TMM in gliomas, the TMM status of a glioma cannot be determined based on genetic signatures alone mainly due to the large number of samples in the negative and both groups.

### Relationship between telomerase activity and age in glioma patients

Investigation of the prognostic value of the TMM considering age and telomerase activity was performed in glioma patients. Among the patients in the telomerase group, the patients with *mutTERTp*, *wtATRX*, or *wtIDH* glioma were significantly older than their counterparts (Additional file 1: Fig. S4). Patients with *mutTERTp* gliomas were also significantly older than those with *wtTERTp* gliomas in the negative and both groups. (Additional file 1: Fig. S4). Irrespective of the TMM group, the RTA in gliomas increased with age, and the number of undetectable RTA cases decreased with age (Additional file 1: Fig. S5a). The age-dependent RTA increase was more prominent at higher WHO grades (Additional file 1: Fig. S5b).

**Fig. 2:**
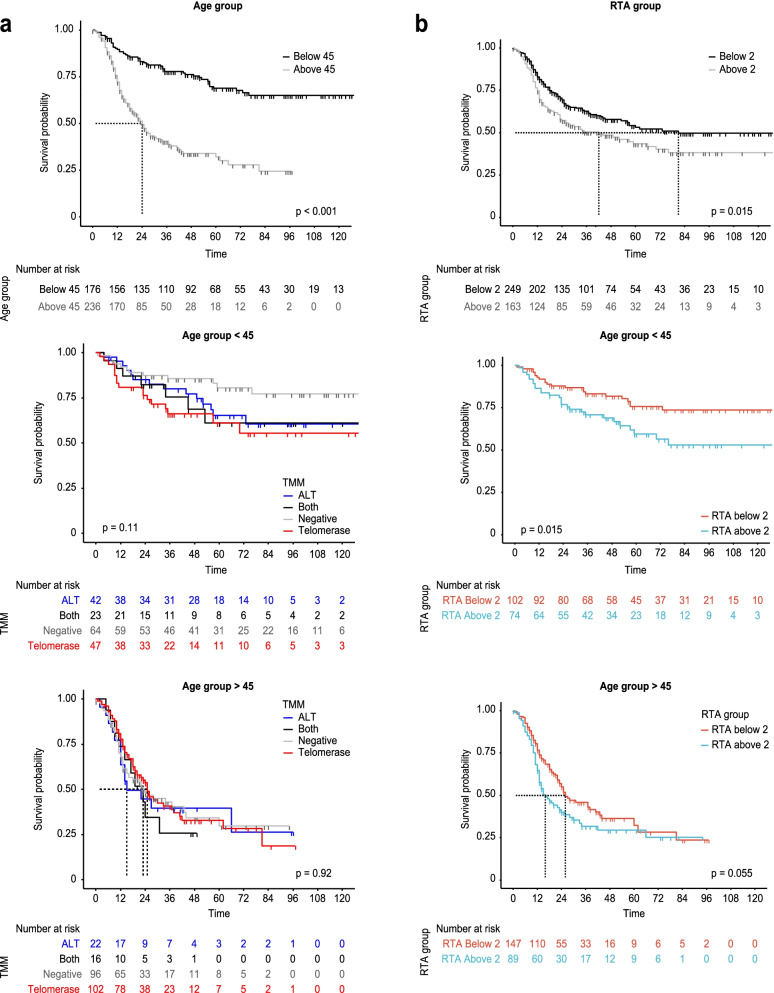
Kaplan-Meier curve of overall survival (OS) in glioma patients (*n* = 412). **a** Comparison of OS in the subsets of patients aged older than and younger than 45 years (top panel, *n* = 236 and *n* = 176, respectively, *p* < 0.0001). Patients younger than 45 years in different telomere maintenance mechanism (TMM) groups (middle panel, *p* = 0.11) and patients older than 45 years in different TMM groups (bottom panel, *p* = 0.92). **b** Comparison of OS in the subsets of patients with relative telomerase activity (RTA) below 2 and above 2 (top panel, *n* = 163 and *n* = 249, respectively, *p* < 0.015). Patients younger than 45 (middle panel, *p* = 0.015) and patients older than 45 (bottom panel, *p* = 0.055) with relative telomerase activity (RTA) below 2 and above 2

There were no differences in the overall survival (OS) among the TMM groups when considering all glioma patients or the subgroup of GBM patients of all ages (Additional file 1: Fig. S6a). To further examine the prognostic effect of telomerase activity according to age, we employed maximally selected rank statistics to determine cutoff points for age (= 45 years) and RTA (= 2) by survival (Additional file 1: Fig. S6b). Patients under the age of 45 showed a significantly more favorable prognosis than patients older than 45 (*p* < 0.0001, Fig. 2a). Again, however, the TMM was not associated with survival differences in either age subgroup (Fig. 2a). Regardless of age, patients with an RTA below 2 showed significantly prolonged survival compared with those with an RTA above 2 (*p* = 0.015, Fig. 2b). This prognostic impact of RTA remained significant in patients aged younger than 45 years (*p* = 0.015, Fig. 2b) and exhibited a trend but did not reach significance in patients aged older than 45 years (*p* = 0.055, Fig. 2b). Prognosis could be successfully stratified by combining the age (45 years) and RTA [2] cutoff criteria in all glioma patients (Additional file 1: Fig. S6c). Multivariate analysis confirmed that RTA below 2 and age below 45 corresponded to significantly low risk (Additional file 1: Fig. S6d). In addition to survival, the age subgroups (with 45 years as the cutoff) shared other distinguishing characteristics, such as TMM distribution (Additional file 1: Fig. S7). The age group below 45 years included mainly cases with lower RTA and a higher proportion of ALT and both TMM cases compared with the older than 45 group. Moreover, the TMM distributions across genetic signatures were different between the age subgroups. For example, *wtATRX/wtTERTp/wtIDH* gliomas were associated with the negative group among patients younger than 45 but closely correlated with the ALT group in patients older than 45. Additionally, *ATRXloss/wtTERTp/mutIDH* gliomas were closely correlated with the both group among patients younger than 45 and with the ALT group among patients older than 45. From the standpoint of the TMM, the age of 45 is a dividing line among gliomas that distinguishes the characteristics of TMM distribution, and telomerase activity itself is a significant prognostic factor even though the TMM group is not.

### Gliomas lacking telomerase activity and ALT (negative group)

**Fig. 3:**
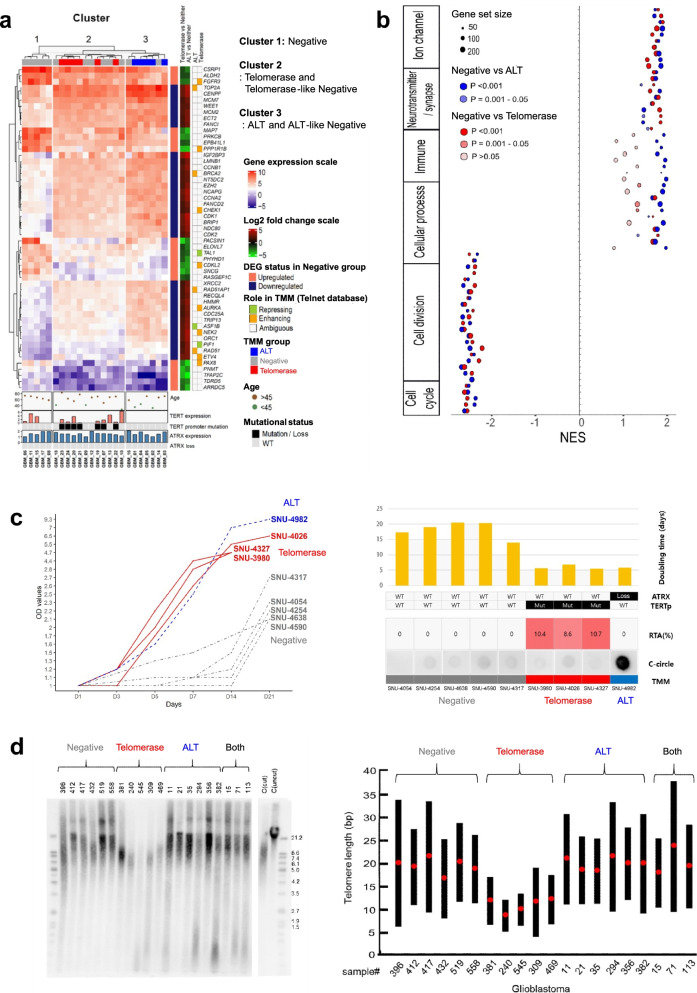
Molecular, signature, and biological characteristics of the negative group. **a** Heatmap with the normalized expression values of 24 TMM samples from RNA-seq data showing 3 distinct clusters in the negative group according to 53 selected significant genes. **b** Dot plot of GSEA preranked analysis showing the topmost significantly enriched functional gene clusters in the negative group compared with the telomerase and ALT groups. Cell cycle- and cell division-related gene sets were negatively enriched in the negative group. **c** TMM characteristics of primary GBM cell lines and differences in their growth rates. Nine primary GBM cell lines were classified into 3 different TMM groups, telomerase (*n* = 3), ALT (*n* = 1), and negative (*n* = 5), according to RTA% and CCA results. Among these nine cell lines, the 5 cell lines categorized as negative showed the slowest growth rates, and their doubling times were longer than those of the telomerase and ALT cell lines. **d** Terminal restriction fragment (TRF) analysis of 20 GBM samples classified into 4 groups: telomerase (*n* = 5), ALT (*n* = 6), both (*n* = 3), and negative (*n* = 6). The telomeres in the telomerase group were shorter than those in the other 3 groups. The telomere lengths of the ALT, both, and negative groups were similar

To investigate the molecular characteristics of gliomas lacking telomerase activity and ALT (negative group), we performed an RNA-seq analysis of 24 GBM samples with various TMMs from the cohort and analyzed the genes involved in telomere maintenance using the TelNet database (http://www.cancertelsys.org/telnet/) [45]. Hierarchical clustering identified 3 clusters of gene expression signatures (cluster 1, negative; cluster 2, telomerase and telomerase-like negative; and cluster 3, ALT and ALT-like negative) categorized by 53 genes that were significantly altered (LFC ≥ 1.5 or ≤ − 1.5) in cluster 1 (negative group) compared with clusters 2 and 3 (Fig. 3a, Additional file 1: Fig. S8, and Additional file 2: Table S1). We validated these 53 selected genes using NanoString analysis (Additional file 2: Table S2) and ultimately defined a set of negative-specific TMM genes that were significantly upregulated (*ALDH2*, *ARRDC5*, *CDKL2*, *CSRP1*, *EPB41L1*, *FGFR3*, *MAP7*, *PACSIN1*, *PHYHD1*, *PPP1R1B*, *PRKCB*, *RASGEF1C*, *SNCG*, *TAL1*, and *TFAP2C*) or downregulated (*IGF2BP3*) by more than 1.5-fold in cluster 1 compared with clusters 2 and 3 in both the RNA-seq and NanoString analysis results (Additional file 1: Fig. S9 and Additional file 2: Table S3). Among these 16 negative-specific TMM genes, only FGFR3 and TAL1 were cataloged by the curated Cancer Gene Census (Additional file 2: Table S4) [49].

We profiled the whole transcriptomes of GBM samples and performed gene set enrichment analysis (GSEA) of the genes that were differentially expressed in the negative group compared with the telomerase and ALT groups (Additional file 2: Table S5). After filtering (false discovery rate (FDR) < 0.0005 and *P* < 0.05), the top 20 functional clusters from each comparison group were identified, and it was found that genes in functional clusters related to cell division and the cell cycle were downregulated and depleted in the negative group (Fig. 3b, Additional file 1: Fig. S10, and Additional file 2: Table S6). This result suggested decreased proliferation activity in the negative group. Consistent with this observation, cell doubling time assays confirmed that the rate of cell division was significantly slower in 5 patient-derived GBM cell lines lacking both telomerase and ALT than in 4 cell lines with telomerase activation or ALT (Fig. 3c). However, there were no differences in the proliferation index (Ki-67) among the TMM groups of clinical GBM samples, except for the significantly higher Ki-67 index in the both group (Additional file 1: Fig. S11).

Telomere length, estimated by terminal restriction fragment (TRF) analysis in 20 randomly chosen representative GBM samples for each TMM group, showed that only the telomerase group had a shorter telomere length than the other groups (Fig. 3d and Additional file 2: Table S7). Telomere length in the negative group exhibited substantial heterogeneity and was similar to that in the ALT and both groups. Collectively, gliomas lacking telomerase activation and ALT are considered to be in an inactive state of proliferation rather than adopting other unknown TMMs to proliferate.

### TMM changes over the course of glioma progression

**Fig. 4:**
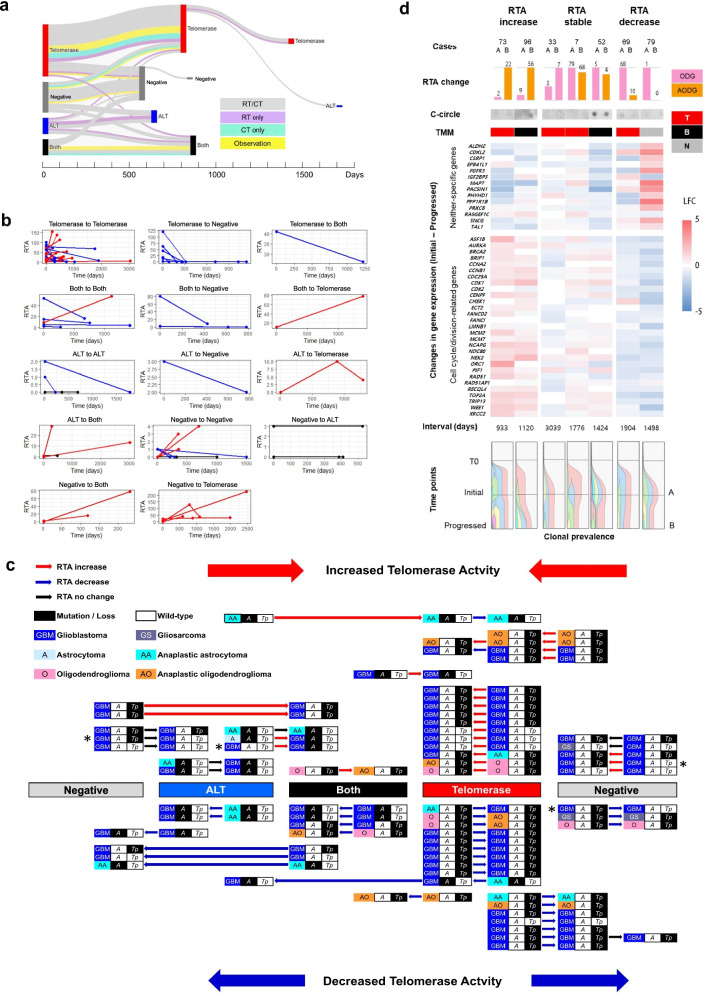
**a** Sankey plot indicating changes in the telomerase maintenance mechanism (TMM) between initial and matched progressed samples. Line colors refer to the treatment conducted. RT, radiotherapy; CT, chemotherapy; Observation, surgery only. The horizontal axis indicates the average progression-free survival time (in days) of patients in each TMM group. **b** Changes in the relative telomerase activity (RTA) under each TMM switching condition. Line colors denote the RTA change (red, increase; blue, decrease; black, no change). **c** Panoramic view of all 64 cases of longitudinal samples showing TMM switching and RTA changes. Four cases (“asterisk”) showed changes in the mutational status of the *TERT promoter* (*Tp*) or *ATRX* loss (*A*) during progression. **d** Changes in the TMM, RTA, and gene expression in 7 cases of natural progression without any intervening treatment between the initial and recurrent samples. Gene expression changes in this switching sample series validated the identified negative-specific TMM genes and cell cycle/division-related genes in cases in the telomerase group with an evident RTA increase. Quantitative clonal evolution diagrams during progression showed an increased number of clones with an RTA increase at progression

We built a separate cohort of 64 glioma patients for whom longitudinal samples were available (133 samples), including 59 pairs and 5 triplets of initial and progressed samples (Additional file 1: Fig. S12a). The median interval between the initial surgery and the second surgery was 538 days (range 83–3039), and the intervals between the second and third surgeries were 303, 315, 402, 880, and 1100 days (Additional file 1: Fig. S12b). We assessed the TMM statuses of the paired samples and classified them in the same manner described above. The TMM status in serial samples was unchanged for 37 patients (57.8%), while the other 27 patients (42.2%) showed switching of the TMM status upon progression (Fig. 4a). The treatment protocol was not a major driver of switching of the TMM (Fig. 4a). Among the patients who showed TMM switching, the switching appeared to occur in a random fashion; we could not identify any specific directionality of TMM switching or any TMM status that was particularly prone to switching. However, we did observe a slightly higher incidence of switching in patients who received treatments compared to the observational category.

When we investigated the changes in RTA, we observed both increases and decreases in the unchanged TMM status categories, while we observed unidirectional changes in RTA within the categories of TMM switching, as expected (Fig. 4b). Based on RTA change directions, TMM classes and all the pairs of samples could be ordered, as shown in Fig. 4c. Telomerase activity was increased with switches in the TMM from the negative to the ALT to the both to the telomerase group, while telomerase activity decreased with switches in the TMM from the telomerase to the both to the ALT to the negative group (Fig. 4c). However, it seemed that the direction of changes in telomerase activity occurred randomly, as cases of increased and decreased RTA showed symmetric configurations (Fig. 4c). Despite RTA changes and TMM switching with glioma progression, the mutational status of *TERTp* and *ATRX* loss remained unchanged in most cases, except in 4 cases of GBM (Fig. 4c). The RTA changes were not influenced by the treatment protocol (Additional file 1: Fig. S13). However, these findings should be considered tentative due to the limited sample size in our longitudinal data.

To verify the genetic and transcriptomic changes in relation to TMM switching without impacts from iatrogenic pressures, we examined patients who did not receive any treatment during the recurrence interval. Nine oligodendroglioma cases progressed naturally without any intervening treatment between the initial and subsequent samples, and 7 of these were available for further analysis. Although all 7 cases remained in the same TMM group after progression, changes in RTA were observed: 2 cases showed an increase in RTA, 3 were relatively stable, and 2 showed a decrease (Fig. 4d). The RNA-seq results again confirmed that the expression levels of negative-specific TMM genes were increased in a sample from the negative group with decreased RTA (case 79) after progression compared with samples from the telomerase or the both group (Fig. 4d, Additional file 2: Table S8). Differential gene expression analysis showed that the expression of cell cycle/division-related genes decreased in general as RTA decreased with progression, whereas the expression increased as RTA increased (Fig. 4d, Additional file 2: Table S9). When we examined clonal evolution using DNA sequencing data, the number of clones increased in progressed samples in which RTA increased (4 to 5 in case 73 and 2 to 4 in case 96), while the number of clones remained the same or even decreased in progressed samples with stable or decreased RTA (Fig. 4d). In summary, the TMM in glioma changes dynamically with tumor progression and reflects the plasticity of the oncogenic biological status of tumor cells.

## Discussion

Before using genetic signatures for surrogate markers for the TMM in gliomas, confirmation of direct evidence of TMM profiles in the whole glioma spectrum should take precedence. We demonstrated that the TMM status in gliomas cannot be simply defined and cannot be predicted by tumor histology or by the presence of gene mutations such as *TERTp* or *ATRX* loss. By measuring the enzymatic activity of telomerase and detecting extrachromosomal telomeric DNA C-circles in glioma tissue samples, we determined that 38.8% of gliomas showed neither telomerase activation nor ALT, while a minor subset of gliomas (9.5%) harbored both telomerase activation and ALT simultaneously (i.e., “both”). The negative group comprised patients of all ages, all histologies, and all grades of gliomas and was not associated with any definitive genetic signatures, whereas the other TMM groups were more likely to have certain genetic signatures, such as the combination of *TERTp/IDH* mutations and *ATRX* loss, and age distributions. However, we detected somatic mutations associated with telomerase activation or ALT in only a subset of gliomas, and their genetic signatures were not perfectly correlated with TMM status; thus, we could not accurately predict TMM group membership on the basis of these mutations. Together, our findings suggest that there are no gene mutations that can consistently be used as markers for telomerase activity or confirm ALT to define the TMM status in gliomas.

It is known that *TERT* expression is not a sufficient surrogate marker for telomerase activity in cancers [17], which has not been clearly characterized in gliomas. Although previous studies have reported that telomerase activity can be estimated on the basis of a group of gene signatures [6, 50], we could not reproduce these results exactly, although we did identify trends among the TMM groups defined by telomerase enzymatic activity measurements. This finding clearly confirms that *TERT* expression is not equivalent to telomerase activation in gliomas.

We observed that significantly more older glioma patients were in the telomerase TMM group than in the other TMM groups. Although the classification of glioma patients by TMM alone failed to show any prognostic significance, the dichotomous cutoff values of age 45 and RTA 2 could successfully discriminate the prognostic groups. Moreover, these results suggest that telomerase activation itself is related to older age and to poor prognosis in gliomas. Studies have confirmed the higher incidence of *TERTp* mutation or *TERT* expression in elderly GBM patients, who are predisposed to telomerase activation [51, 52]. Although its relationship with patient survival has been controversial, *TERTp* mutation is frequently associated with poor OS in patients with all subtypes of glioma [53–55]. It is recognized that *TERT* can become prone to reactivation with aging and progressive telomere shortening, increasing the risk of cancer [56]. Indeed, in this study, it was observed that the GBMs in the telomerase group which were more common among older patients, tended to have shorter telomeres than the GBMs in the other TMM groups. These findings are consistent with the hypothesis that the oncogenesis of gliomas with telomerase activation is associated with age-dependent telomere shortening, which perturbs the tightly regulated expression of telomerase in human cells.

Another highlight of the present investigation of the TMM classification of glioma is improved understanding of the biological behaviors of glioma cells in the negative group and their underlying mechanisms. By analyzing the differential expression of genes listed in the TelNet database among TMM groups and validating these differences with the NanoString method, we were able to define 16 negative-specific TMM genes. One of the initial studies on telomerase activity and ALT in GBM showed that among 77 GBM patients, the largest TMM subgroup (47%) did not show ALT or telomerase activity, followed by the subgroups with telomerase activity (29%), ALT (19%), and both (5%) [16]. There is sound evidence that some cancer types lack both telomerase activity and ALT [57–59]. A pan-cancer study using more than 18,000 cancer samples demonstrated that 22% did not express either *TERT* or ALT-associated alterations [6]. It remains unclear whether these cancers have a third unknown TMM. In the present study, it was difficult to deduce whether TMMs other than telomerase activation or ALT might occur based on the functional annotation of the negative-specific TMM genes. However, the downregulation of genes related to the cell cycle or cell division was consistently enriched in the negative group. Moreover, in vitro experiments using GBM tissue and patient-derived cell lines revealed that the telomeres in the negative group were longer and more heterogeneous in length than those in the telomerase group and that these cells exhibited a fairly slow growth rate. This type of tumor is therefore distinct from the so-called “ever-shorter telomeres” type of tumor, which is characterized by lengthy telomeres and the lack of a TMM but extensive proliferation capacity and aggressive behavior [60]. Despite this result, we did not observe a difference in overall survival between the negative group and the other TMM groups. Thus, there may be an unknown temporal cellular dynamic during cancer progression that maintains the oncologic potential of this class of tumors.

Switching of the TMM, potentially concomitant with transcriptional reprogramming, during the course of the disease is one hypothesis for how tumors with this profile are able to proliferate and invade. Our longitudinal data from glioma samples demonstrated that the switching of TMM groups or changes in telomerase activity are common scenarios in glioma progression, although the mutational status of *TERTp* and *ATRX* loss are mostly unchanged. The existence of the both group in this study reflects TMM dynamics during glioma progression; there is also considerable evidence that telomerase activation and ALT can coexist within a single tumor or may undergo a mutual shift during cancer progression in relation to anticancer therapy or EMT [19–25, 61–64]. For example, previous in vitro and in vivo experiments showed that cancer cells with activated telomerase converted to an ALT profile after telomerase inhibition [19–22]. Additionally, telomerase reactivation causes cancer progression and aggressive cancer behavior [21, 65], which are preceded by an increase in clonal heterogeneity and subsequent selection [66]. Here, we also observed an increased complexity of clonal evolution dynamics in gliomas with increased telomerase activity during progression. These observations show that the TMM status in gliomas is not innate and immutable but changes continuously under internal or external pressures to promote oncogenesis. Interestingly, to our knowledge, whether telomerase activity increases or decreases with disease progression currently appears to be random. However, despite intriguing findings regarding TMM status in gliomas, our study does have some considerable limitations, such as using samples from a single institution with mostly Asian samples. Future research similar to our study with diverse and large samples is needed to understand the pressures that drive TMM switching. Moreover, the effect of TMM dynamics on prognosis still needs to be fully addressed in the future to develop TMM as a target for anticancer strategies.

## Conclusions

In conclusion, the TMM status in gliomas cannot be determined simply on the basis of underlying telomere-related genetic signatures; therefore, future researchers should also consider defining this status by directly measuring telomerase enzyme activity and ALT and should not limit TMM by representing ATRX or TERT mutation as definitive indicators. Considerable proportions of gliomas lack both telomerase activity and ALT, and the TMM is dynamic and can be switched during glioma progression, reflecting the plasticity of the oncogenic biological status of tumor cells. Future work focusing on TMM dynamics in glioma progression is expected to uncover crucial biological pathways that can be used in novel therapeutic strategies.

## Supplementary Information

**Additional file 1:** Figs. S1–S13.

**Additional file 2:** Tables S1–S9.

## Data Availability

All data are published within this paper and accompanying additional files (indicated in the text) and can be accessed via weblink on the journal site. The raw data for RNA-seq and WES used in this study have been submitted to the Short-Read Sequence Archive (SRA) under the accession number PRJNA825658 (https://www.ncbi.nlm.nih.gov/bioproject/PRJNA825658) [67].

## Abbreviations

TMMs: Telomere maintenance mechanisms
ALT: Alternative lengthening of telomeres
TERT: Telomerase reverse transcriptase
TRAP: Telomerase repeated amplification protocol
CCA: C-circle assay
RTA: Relative telomerase activity
