# Supplementary material for: The telomere maintenance mechanism spectrum and its dynamics in gliomas
Source: Genome Med. 2022 Aug 11;14:88. doi: 10.1186/s13073-022-01095-x (PMC9367055; doi:10.1186/s13073-022-01095-x)

Figure S1 a. Distribution of patients according to age (ranging from 6 months to 82 years). **b.** Pie chart showing the distribution of histological diagnoses among 412 glioma patients. **c**. Telomerase maintenance mechanism (TMM) groups were defined based on relative telomerase activity (RTA) and c-circle assay (CCA) results. The result of CCA was considered positive if the quantified relative value of the blot intensity >30. RTA was calculated by dividing telomerase activity by heated telomerase activity in the telomerase repeated amplification protocol (TRAP). The Telomerase group includes patients with evident telomerase activity (RTA ≥ 2) without any ALT activity (negative CCA), while the ALT group includes patients without evident telomerase activity (RTA<2) and with ALT activity (positive CCA). The Both group included patients with evident telomerase activity and ALT (RTA ≥ 2 and positive CCA), while the Negative group included patients with limited evidence of telomerase activity and ALT (RTA<2 and negative CCA). **d.** The subdistribution of histological diagnosis among TMM groups. **e.** The subdistribution of TMM groups among gliomas grouped according to histological diagnosis. (GBM; glioblastoma, GS; gliosarcoma, DMG; diffuse midline glioma, AA; anaplastic astrocytoma, A; astrocytoma, AODG; anaplastic oligodendroglioma, ODG; oligodendroglioma, PA; pilocytic astrocytoma, PXA; pleomorphic xanthoastrocytoma, SEGA; subependymal giant cell astrocytoma)

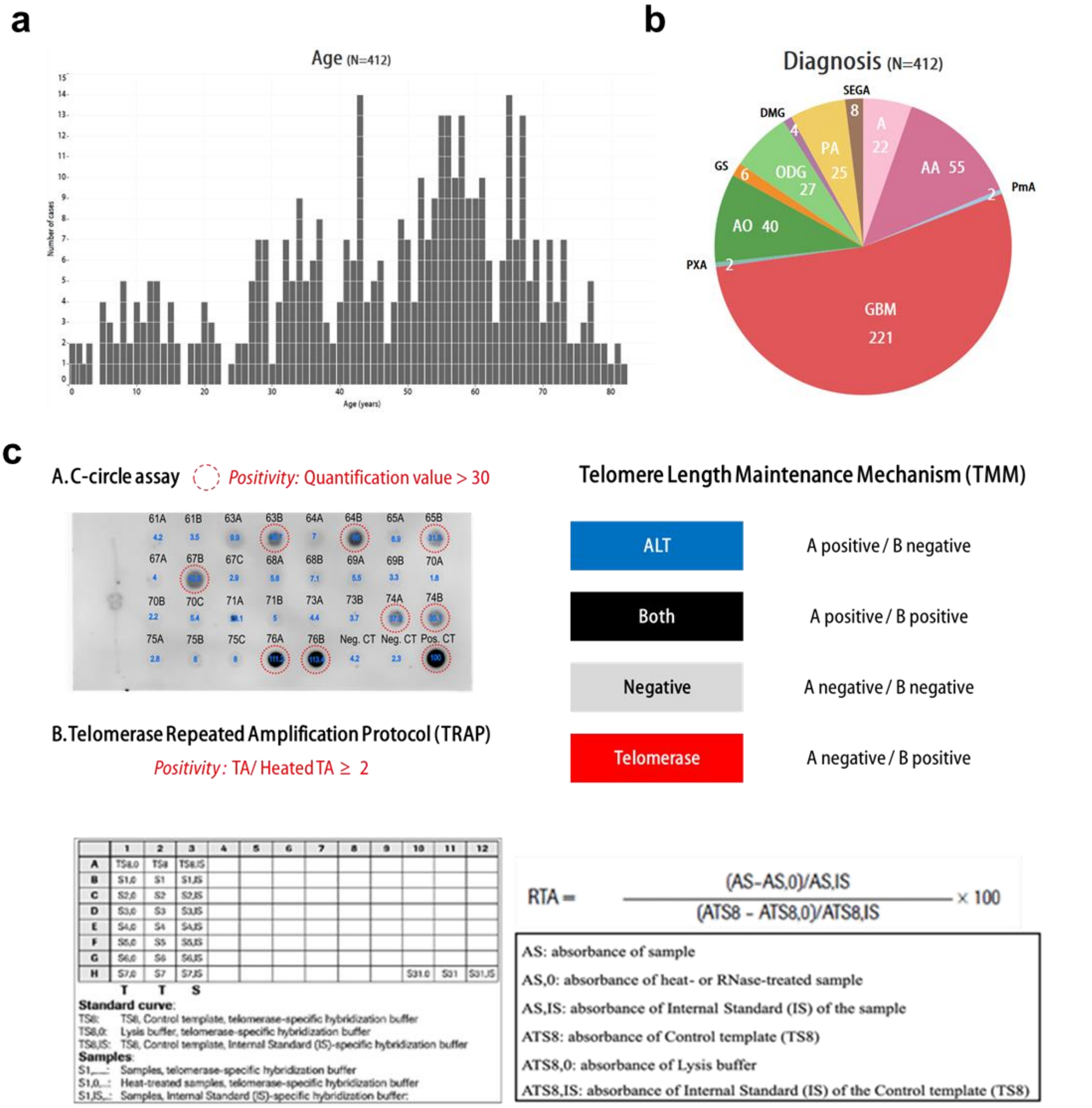

d

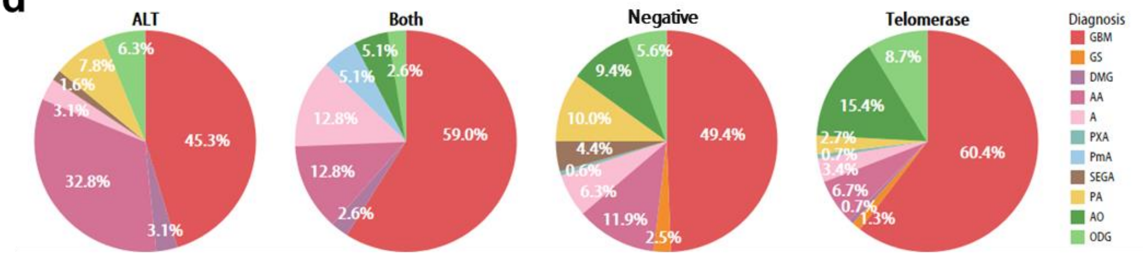
ALT
45.3%
32.8%
3.1%
3.1%
1.6%
7.8%
6.3%
Both
59.0%
12.8%
12.8%
2.6%
5.1%
5.1%
2.6%
Negative
49.4%
2.5%
11.9%
6.3%
0.6%
4.4%
10.0%
9.4%
5.6%
Telomerase
60.4%
1.3%
0.7%
6.7%
3.4%
0.7%
2.7%
15.4%
8.7%
Diagnosis
GBM
GS
DMG
AA
A
PXA
PmA
SEGA
PA
AO
ODG

e

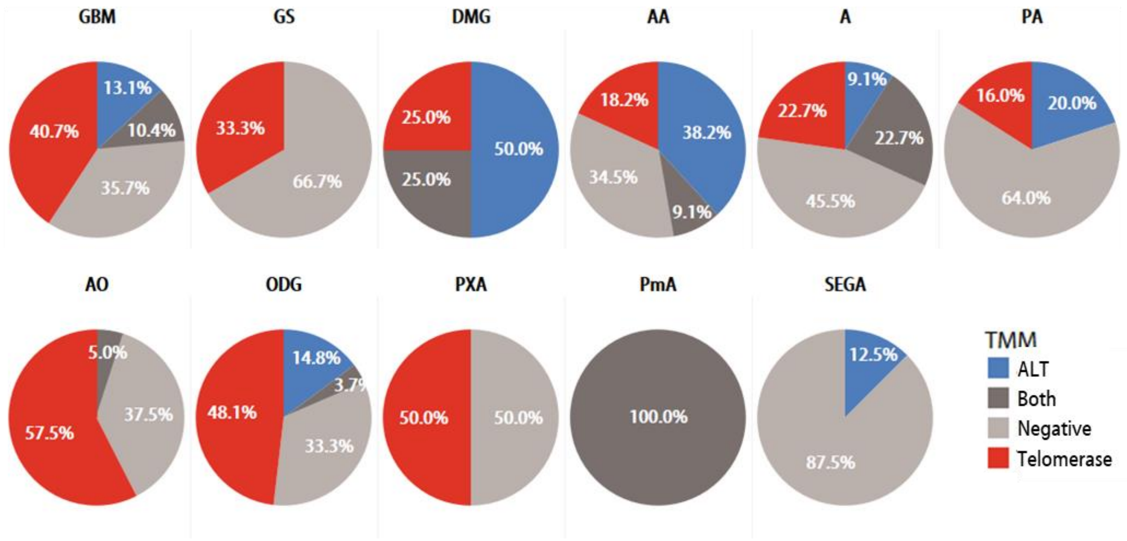
GBM
13.1%
10.4%
35.7%
40.7%
GS
33.3%
66.7%
DMG
50.0%
25.0%
25.0%
AA
38.2%
9.1%
34.5%
18.2%
A
9.1%
22.7%
45.5%
22.7%
PA
20.0%
64.0%
16.0%
AO
5.0%
37.5%
57.5%
ODG
14.8%
33.3%
48.1%
PXA
50.0%
50.0%
PmA
100.0%
SEGA
12.5%
87.5%
TMM
ALT
Both
Negative
Telomerase

Figure S2 The genetic and clinical characteristics of glioma patients in each TMM group. Note that the *TERTp* and *ATRX* loss are mutually exclusive but are not closely correlated with the TMM. **a.** Telomerase group (n=149). **b.** Negative group (n=160). **c**. ALT group (n=64). **d.** Both group (n=39)

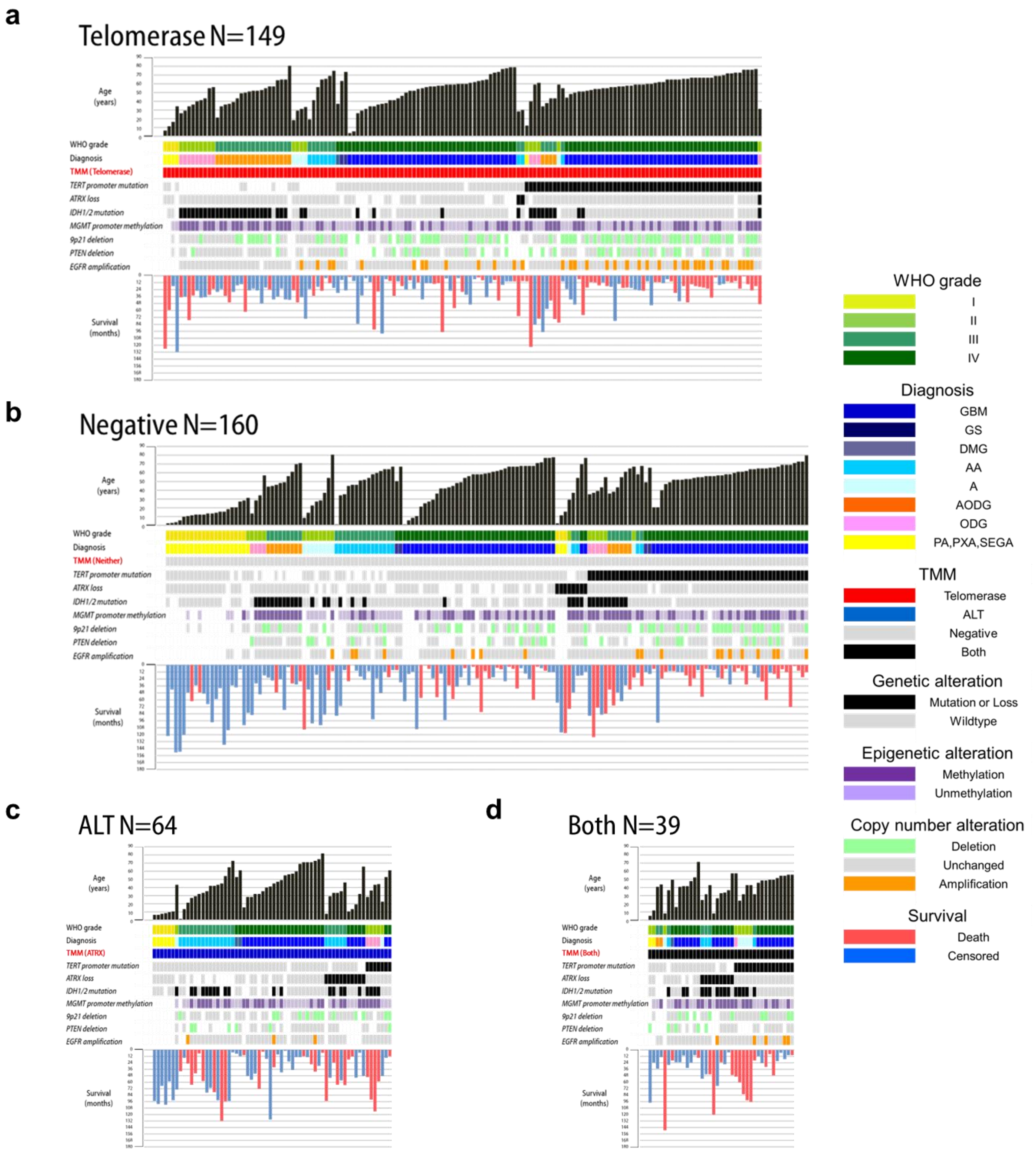

Figure S3 a. TMM status according to *TERTp* and *ATRX* loss statuses. The correlogram shows a strong positive correlation between the ALT group and *mutATRX/wtTERTp* or *wtATRX/wtTERTp* gliomas and a strong negative correlation between the ALT group and *wtATRX/mutTERTp* gliomas. The Both group had a strong positive correlation with *mutATRX/wtTERTp* gliomas and a negative correlation with *wtATRX/wtTERTp* gliomas. The Telomerase group had a strong negative correlation with *mutATRX/wtTERTp* gliomas and a positive correlation with *wtATRX/mutTERTp* gliomas. Note that the Negative group had no significant correlation with the *TERTp* or *ATRX* status. There was a single unusual case harboring mutation in both *TERTp* and *ATRX.* The color intensity and size of the circle are proportional to the correlation coefficients. Positive correlations are displayed in blue, while negative correlations are displayed in red (Pearson residuals, chi-squared = 36.848, df = 9, p<0.001). **b.** TMM status according to *IDH1* and *IDH2* mutation status. The correlogram indicates that *mutIDH* has strong positive correlations with the ALT and Both groups and a strong negative correlation with the Negative group (Pearson residuals, chi-squared = 7.9992, df = 3, p=0.046). **c.** TMM, *ATRX, EGFR, PTEN* status according to *TERTp* mutation status. 32.8% and 10.9% *TERTp mutation samples had* EGFR amplification and PTEN deletion respectively, 7.9% and 5.6% *TERTp wild type samples had* EGFR amplification and PTEN deletion respectively. *TERTp* mutation samples were mostly classified as Telomerase group with some Negative and Both, *TERTp* wild type samples had all 4 TMM groups.

a

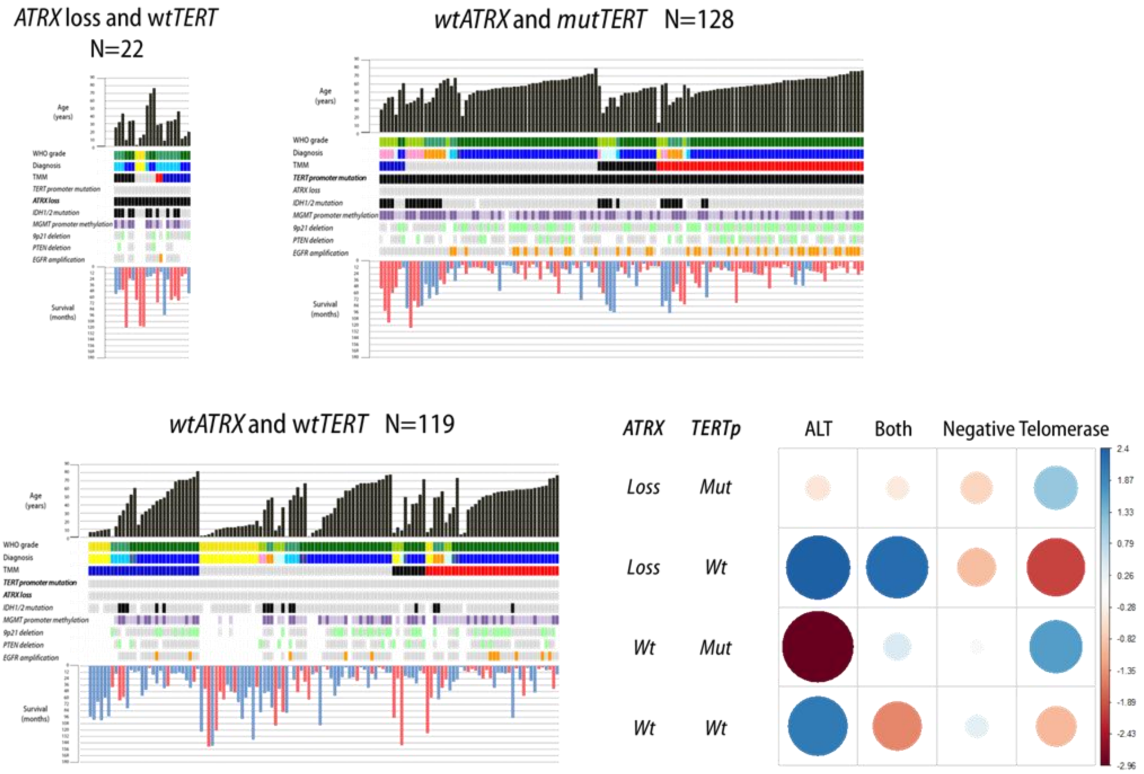

b

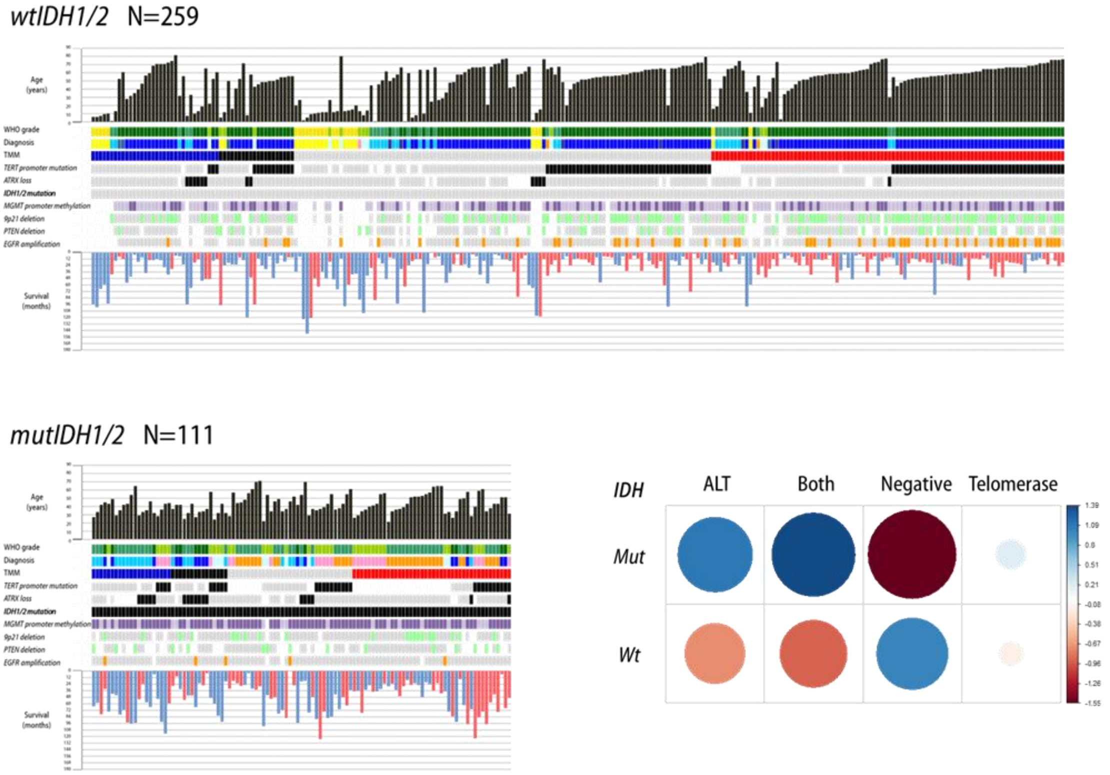
wtIDH1/2 N=259
Age (years)
WHO grade
Diagnosis
TMM
TERT promoter mutation
ATRX loss
IDH1/2 mutation
MGMT promoter methylation
9p21 deletion
PTEN deletion
EGFR amplification
Survival (months)
mutIDH1/2 N=111
IDH
ALT
Both
Negative
Telomerase
Mut
Wt

c

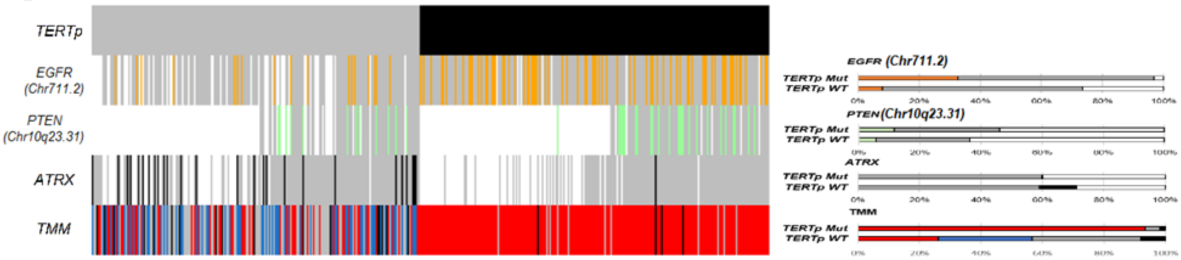
TERTp
EGFR (Chr711.2)
PTEN (Chr10q23.31)
ATRX
TMM
TERTp Mut
TERTp WT

Figure S4 Comparison of age distributions in TMM groups stratified according to major signature gene status (Student's t-test; *p<0.05, **p<0.01, ***p<0.001). **a.** *TERT promoter*. **b.** *ATRX*. **c**. *IDH*. **d.** *MGMT promoter*. (Mut; mutation, WT. wild-type, M; methylated, U; unmethylated)

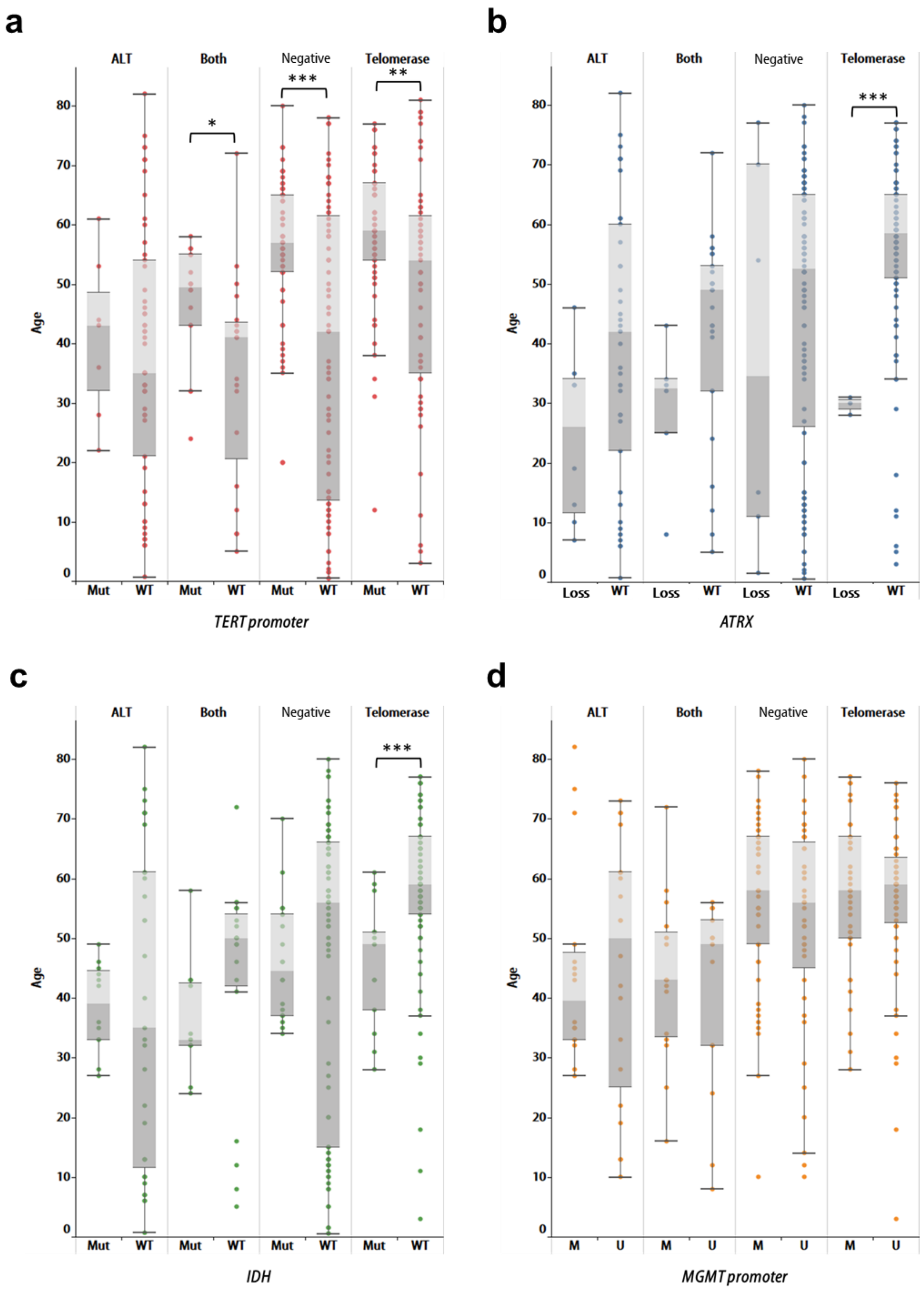

Figure S5 a. Relative telomerase activity (RTA) trends with age. More patients with detectable RTA and fewer patients with undetectable RTA are observed with increasing age. **b.** Age-dependent RTA distributions among TMM groups and WHO grades.

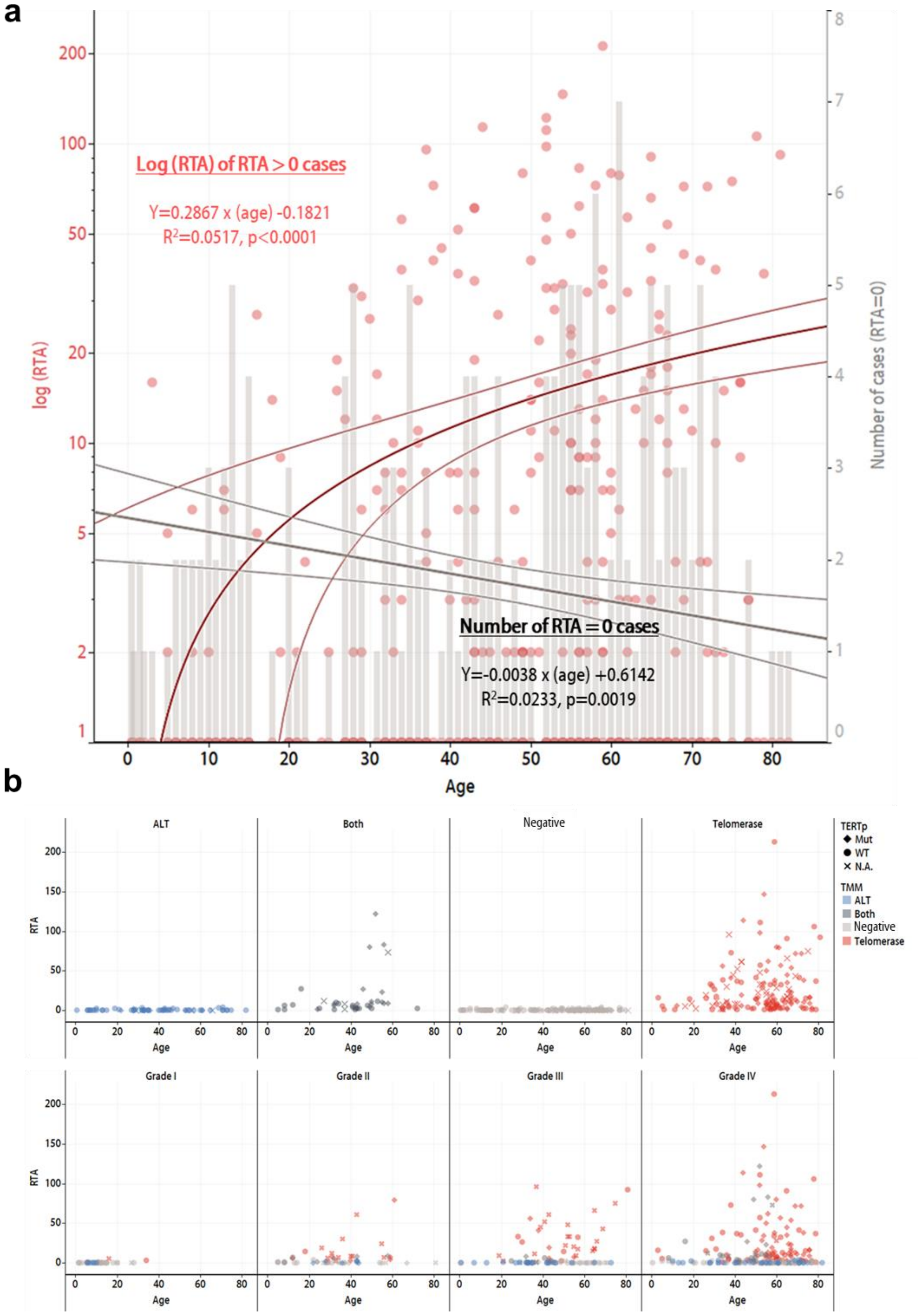

Figure S6 a. Kaplan-Meier curve of overall survival (OS) in all glioma patients stratified by TMM group (p=0.22). No significant differences in OS were observed among the TMM groups. **b.** Optimal cutoffs of age (age 45 years) and relative telomerase activity (RTA 2) for OS were determined by means of maximally selected log-rank statistics. **c.** The application of optimal age and RTA cutoffs for defining prognostic groups of glioma patients was confirmed by Kaplan-Meier analysis (p<0.0001). **d.** Cox proportional hazards model of TMM, age, RTA, grade and IDH mutation showing significant low risk in RTA ≤2 and age below 45 groups.

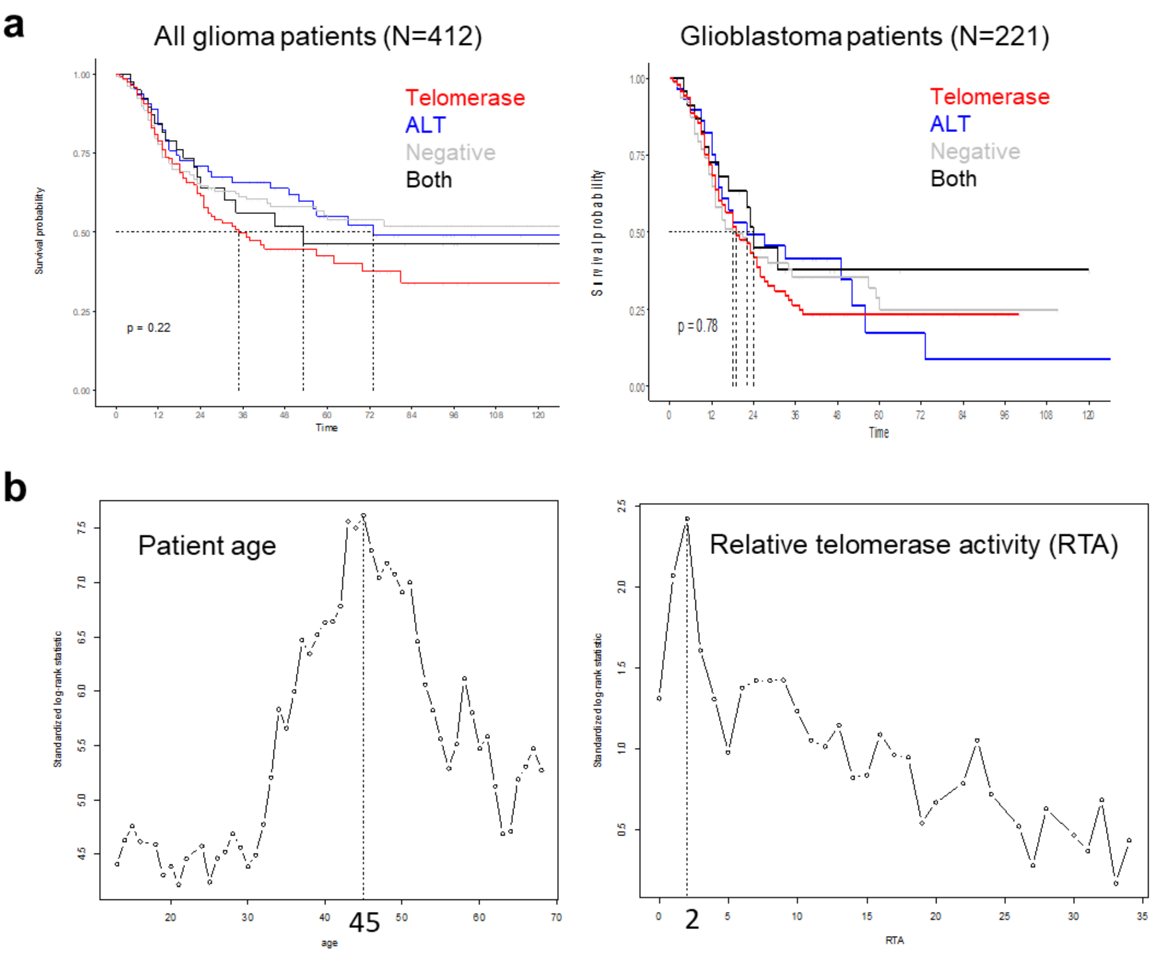

c

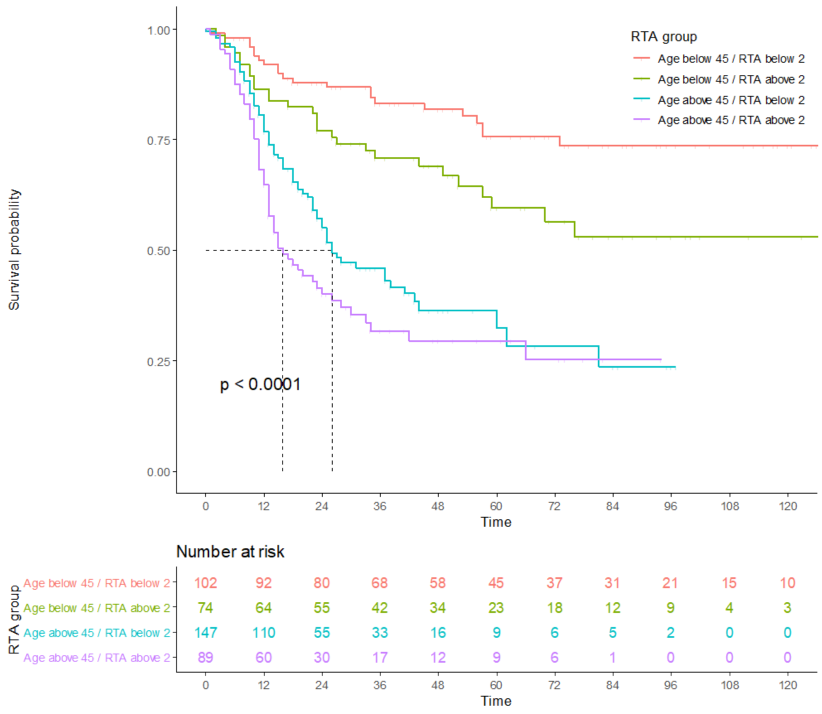
RTA group
Age below 45 / RTA below 2
Age below 45 / RTA above 2
Age above 45 / RTA below 2
Age above 45 / RTA above 2
Survival probability
1.00
0.75
0.50
0.25
0.00
p < 0.0001
0 12 24 36 48 60 72 84 96 108 120
Time
Number at risk
RTA group
Age below 45 / RTA below 2 102 92 80 68 58 45 37 31 21 15 10
Age below 45 / RTA above 2 74 64 55 42 34 23 18 12 9 4 3
Age above 45 / RTA below 2 147 110 55 33 16 9 6 5 2 0 0
Age above 45 / RTA above 2 89 60 30 17 12 9 6 1 0 0 0
0 12 24 36 48 60 72 84 96 108 120
Time

d

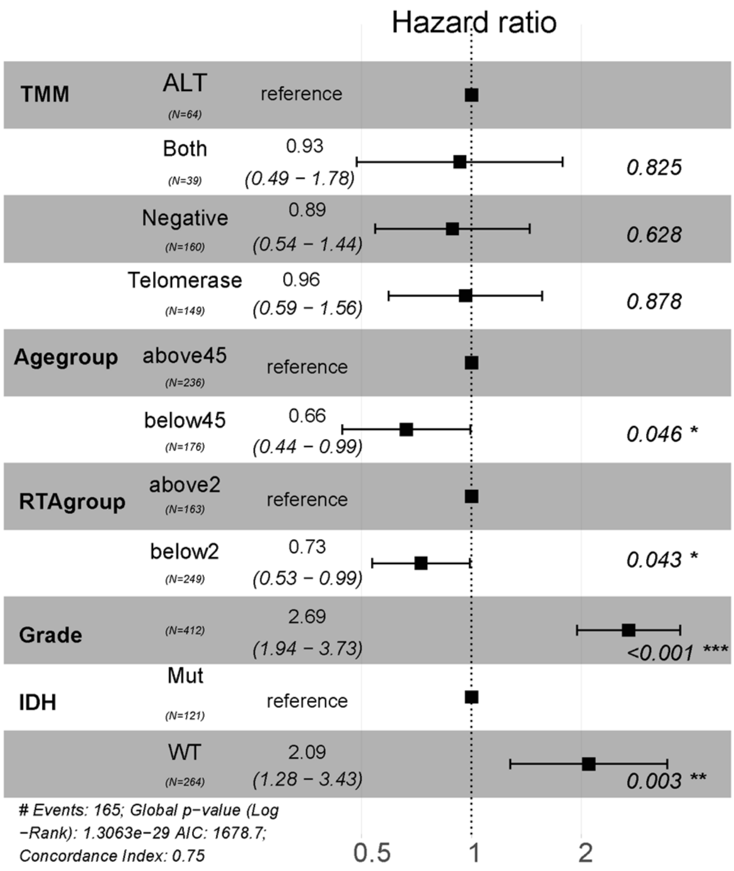
Hazard ratio
TMM ALT (N=64) reference
Both (N=39) 0.93 (0.49 − 1.78) 0.825
Negative (N=160) 0.89 (0.54 − 1.44) 0.628
Telomerase (N=149) 0.96 (0.59 − 1.56) 0.878
Agegroup above45 (N=236) reference
below45 (N=176) 0.66 (0.44 − 0.99) 0.046 *
RTAgroup above2 (N=163) reference
below2 (N=249) 0.73 (0.53 − 0.99) 0.043 *
Grade (N=412) 2.69 (1.94 − 3.73) <0.001 ***
IDH Mut (N=121) reference
WT (N=264) 2.09 (1.28 − 3.43) 0.003 **
Events: 165; Global p−value (Log −Rank): 1.3063e−29 AIC: 1678.7; Concordance Index: 0.75
0.5
1
2

Figure S7 The distribution of TMM groups in age subsets, additionally stratified by major genetic signatures in glioma and their correlogram. The color intensity and size of the circle are proportional to the correlation coefficients. **a.** Subset of patients aged younger than 45 (Pearson residuals, chi-squared = 25.269, df = 15, p=0.04644). **b.** Subset of patients aged older than 45 (Pearson residuals, chi-squared = 28.18, df = 15, p=0.02047)

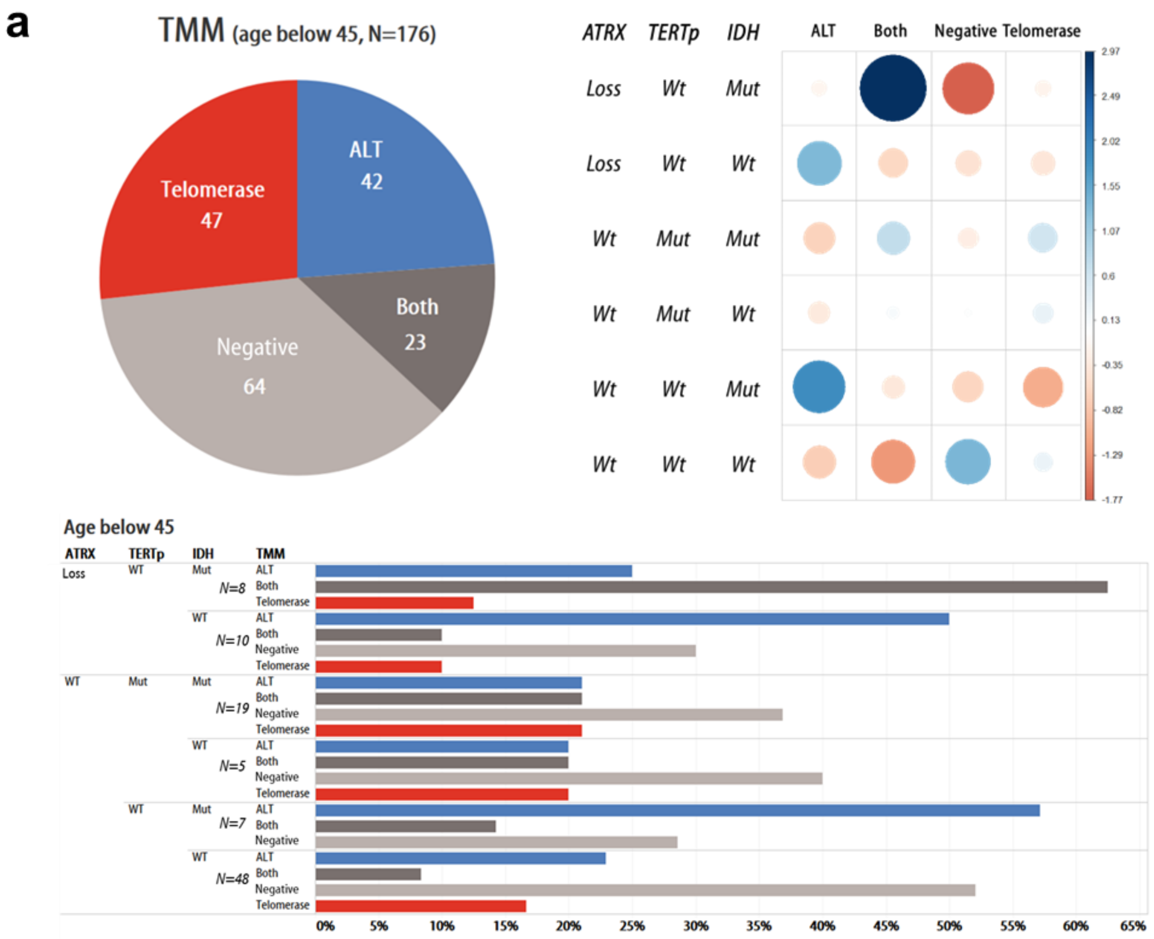

b

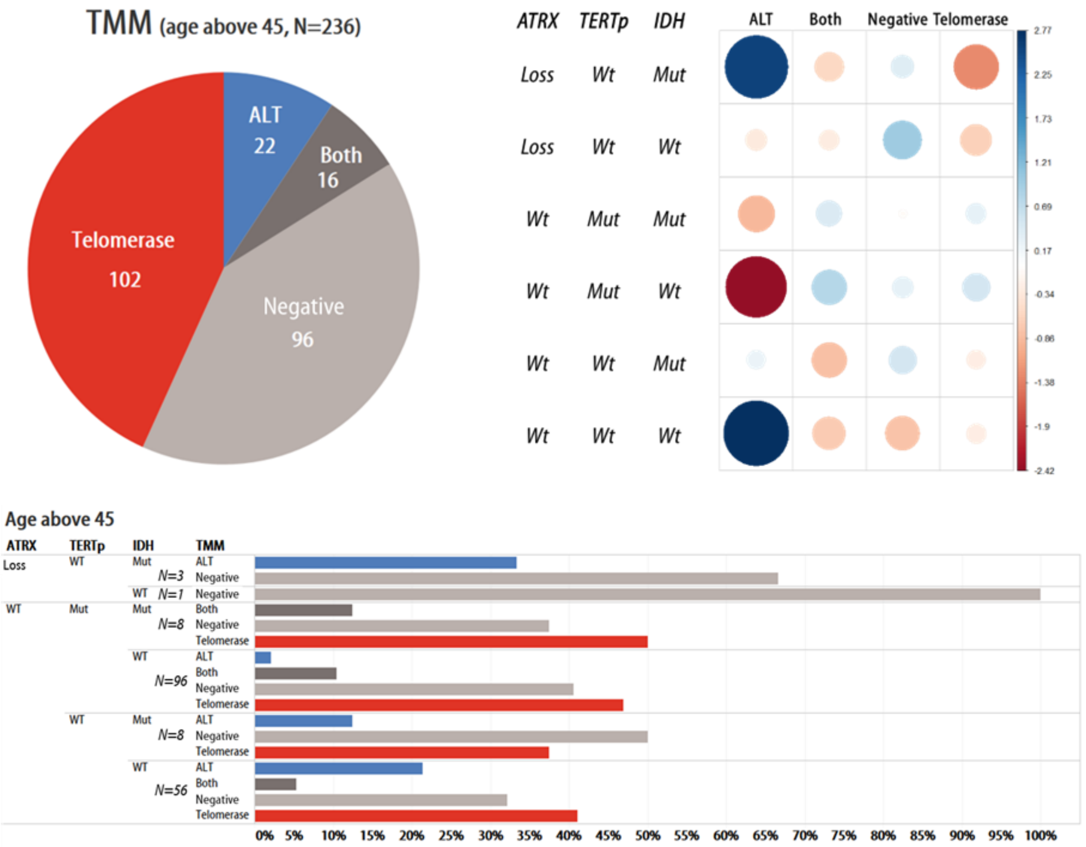
TMM (age above 45, N=236)
ALT
22
Both
16
Telomerase
102
Negative
96
ATRX
TERTp
IDH
ALT
Both
Negative
Telomerase
Loss
Wt
Mut
Loss
Wt
Wt
Wt
Mut
Mut
Wt
Mut
Wt
Wt
Wt
Mut
Wt
Wt
Wt
2.77
2.25
1.73
1.21
0.69
0.17
-0.34
-0.86
-1.38
-1.9
-2.42
Age above 45
ATRX
TERTp
IDH
TMM
Loss
WT
Mut
N=3
ALT
Negative
WT
N=1
Negative
WT
Mut
Mut
N=8
Both
Negative
Telomerase
WT
N=96
ALT
Both
Negative
Telomerase
WT
Mut
N=8
ALT
Negative
Telomerase
WT
N=56
ALT
Both
Negative
Telomerase
0%
5%
10%
15%
20%
25%
30%
35%
40%
45%
50%
55%
60%
65%
70%
75%
80%
85%
90%
95%
100%

Figure S8 PCA plot showing three distinct clusters of 24 GBM samples according to 53 TMM-related genes. Clusters 1, 2 and 3 comprise Negative, Telomerase and Telomerase-like Negative, and ALT and ALT-like Negative samples, respectively

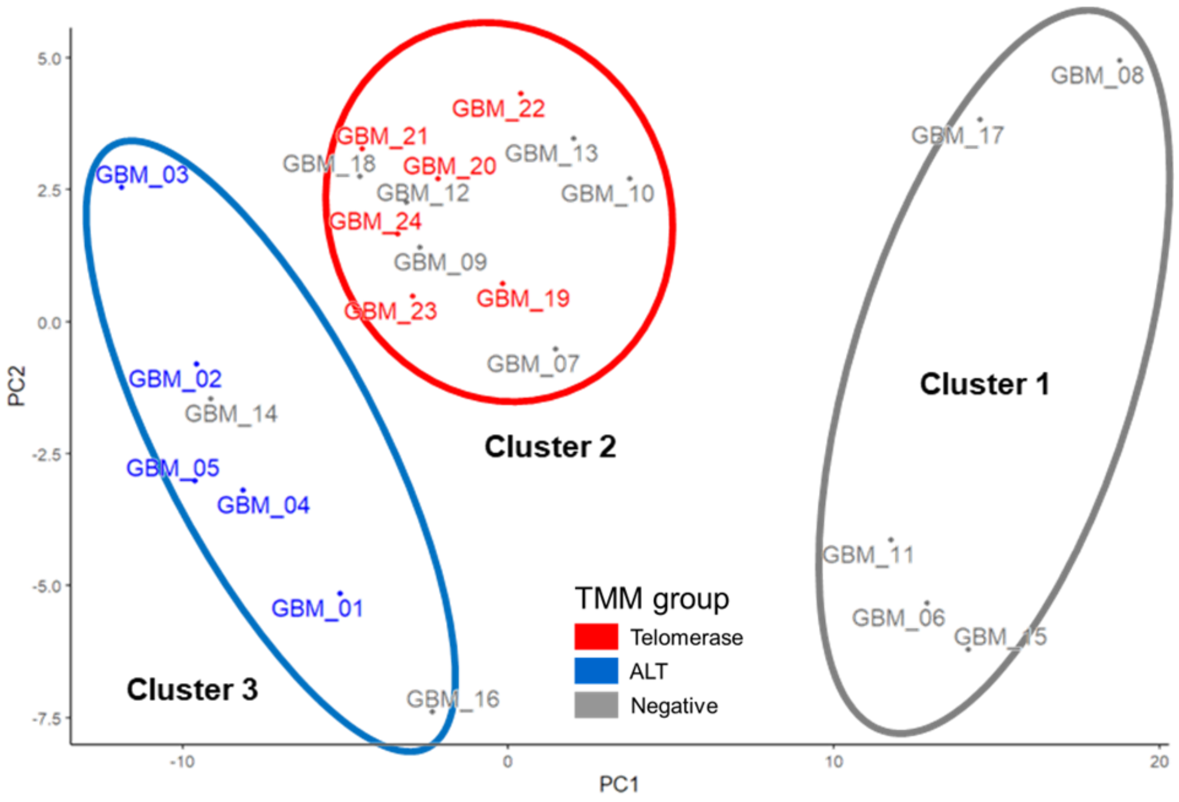

Figure S9 NanoString validation of Negative-specific genes. **a.** Correlation plot of RNA-seq data with NanoString data for Cluster 1 vs Clusters 2 and 3 showing 16 genes that were validated as Negative-specific. **b.** Box plots of NanoString expression data for16 Negative-specific genes that were significantly differentially expressed in Cluster 1 compared with Clusters 2 and 3

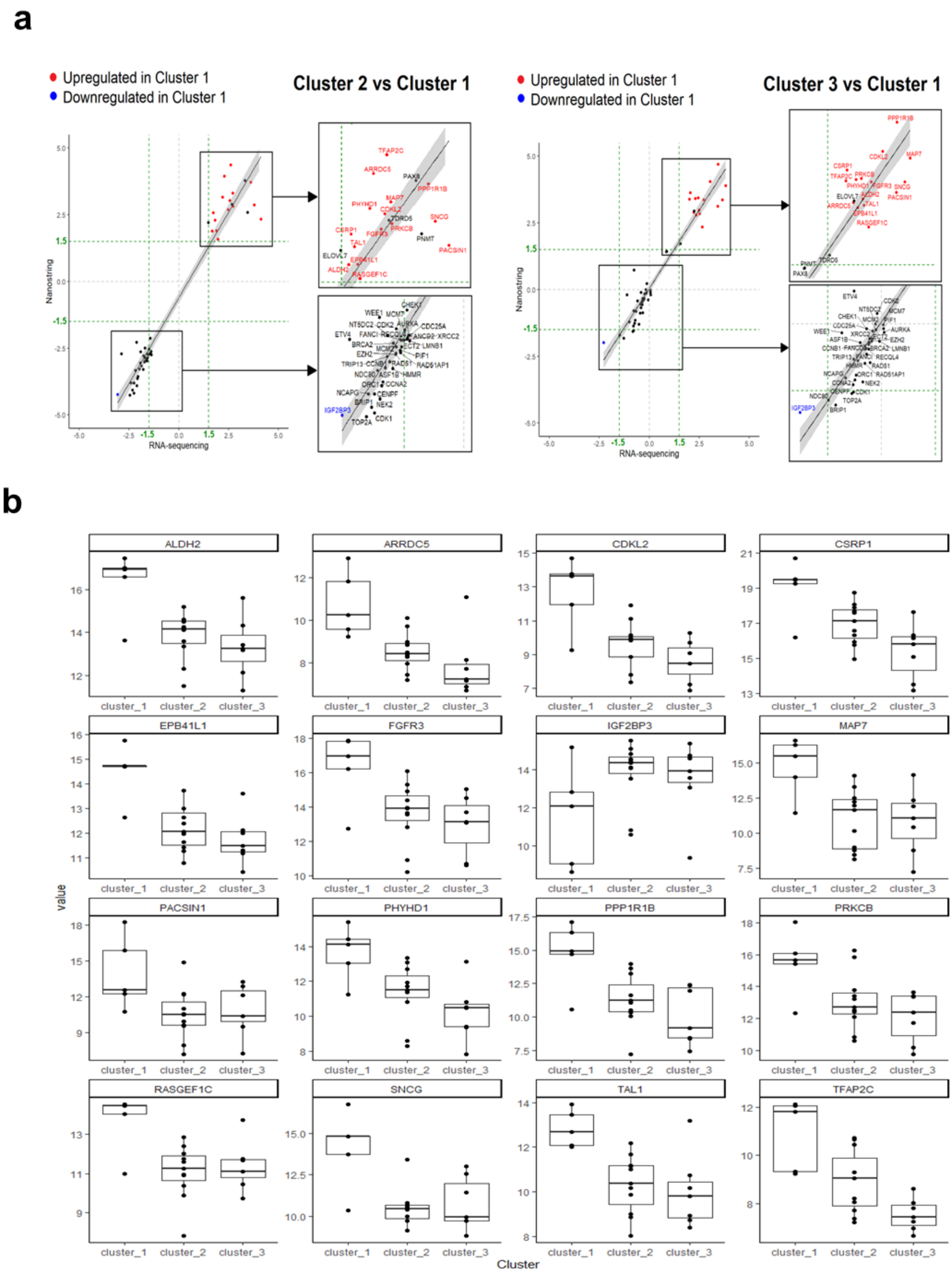

Figure S10 Detailed dot plot of the GSEA preranked analysis showing the gene set names in each significant functional group. Cell cycle- and cell division-related genes were downregulated in the Negative group compared to the Telomerase and ALT groups

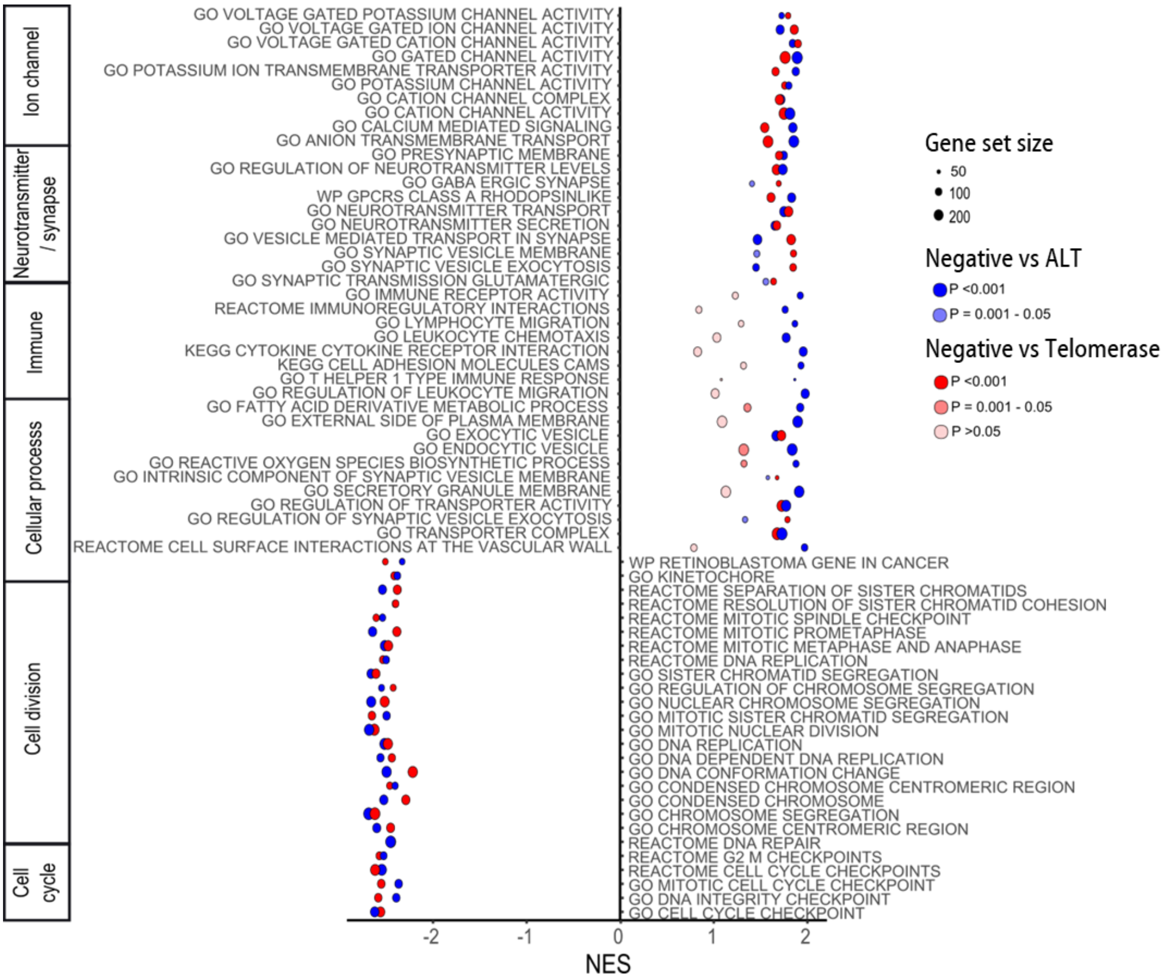

Figure S11 Comparison of the proliferation index (Ki-67) of glioblastoma tissue samples (n=221) among TMM groups (ANOVA with the Bonferroni correction T-test; *p<0.05, **p<0.01). The Both group had a significantly higher Ki-67 index than the other groups

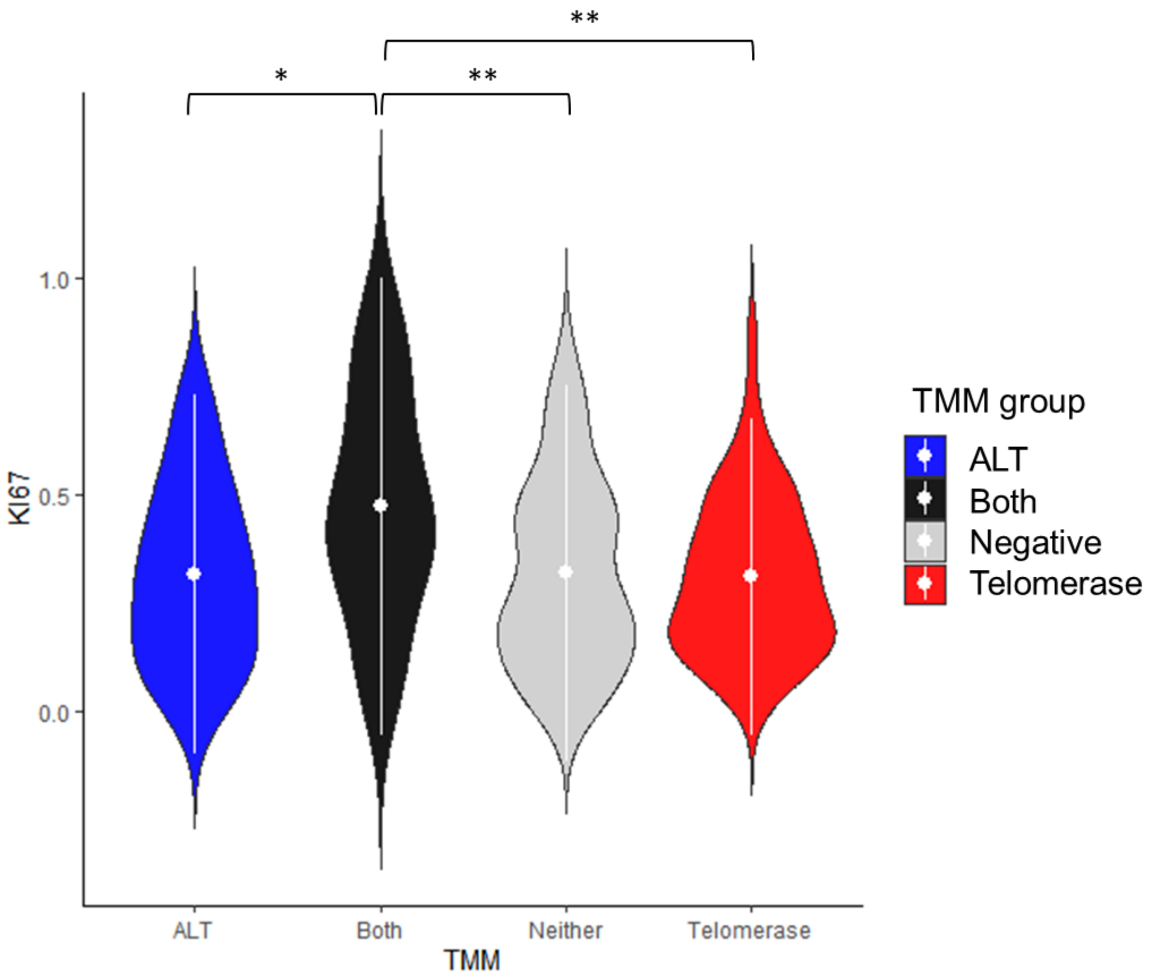

Figure S12 a. Summary of 64 glioma patients with longitudinal samples. Abbreviations indicate the histological diagnosis (O, oligodendroglioma; AO, anaplastic oligodendroglioma; A, astrocytoma; AA, anaplastic astrocytoma; GBM, glioblastoma; GS. i). **b.** Distribution of recurrence intervals between the initial tumor and first recurrence (interval 1) and between the first and second recurrences (interval 2)

a

Primary		Recurrent1		Recurrent2	Number of patients
O	→	O			2
O	→	AO			5
AO	→	AO	→	(AO)	4 (1)
A	→	GBM			1
AA	→	AA	→	(AA)	4 (1)
AA	→	GBM	→	(GBM)	6 (1)
GBM	→	GBM	→	(GBM)	40 (2)
GBM	→	GS			1
GS	→	GS			1

b

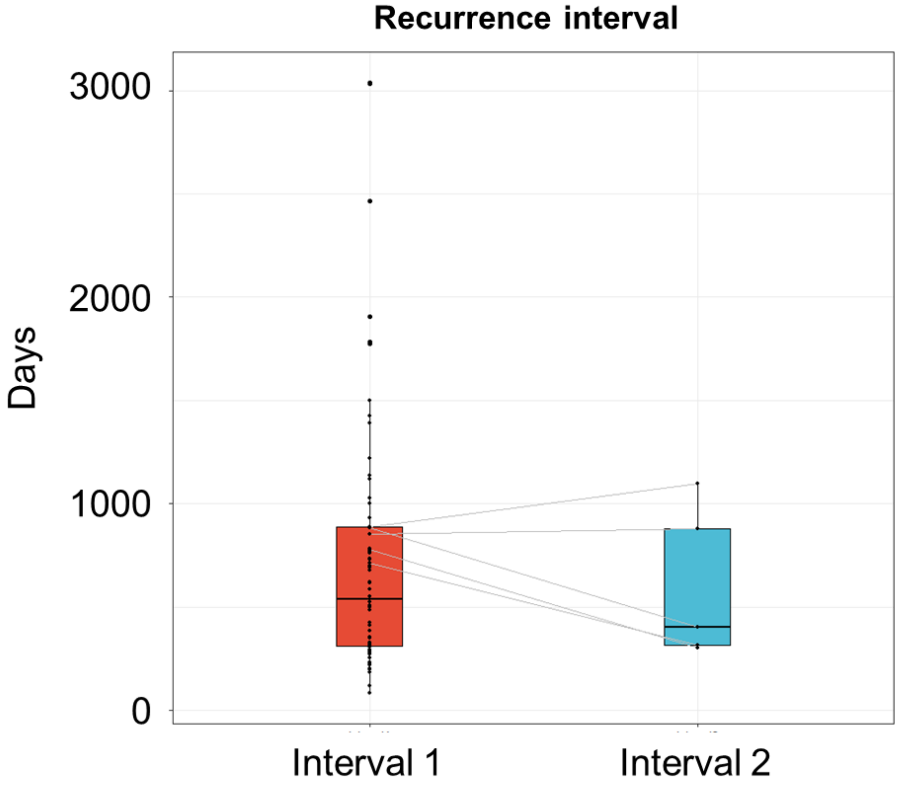

Figure S13 Distribution of patients with RTA changes according to the initial treatment protocol.

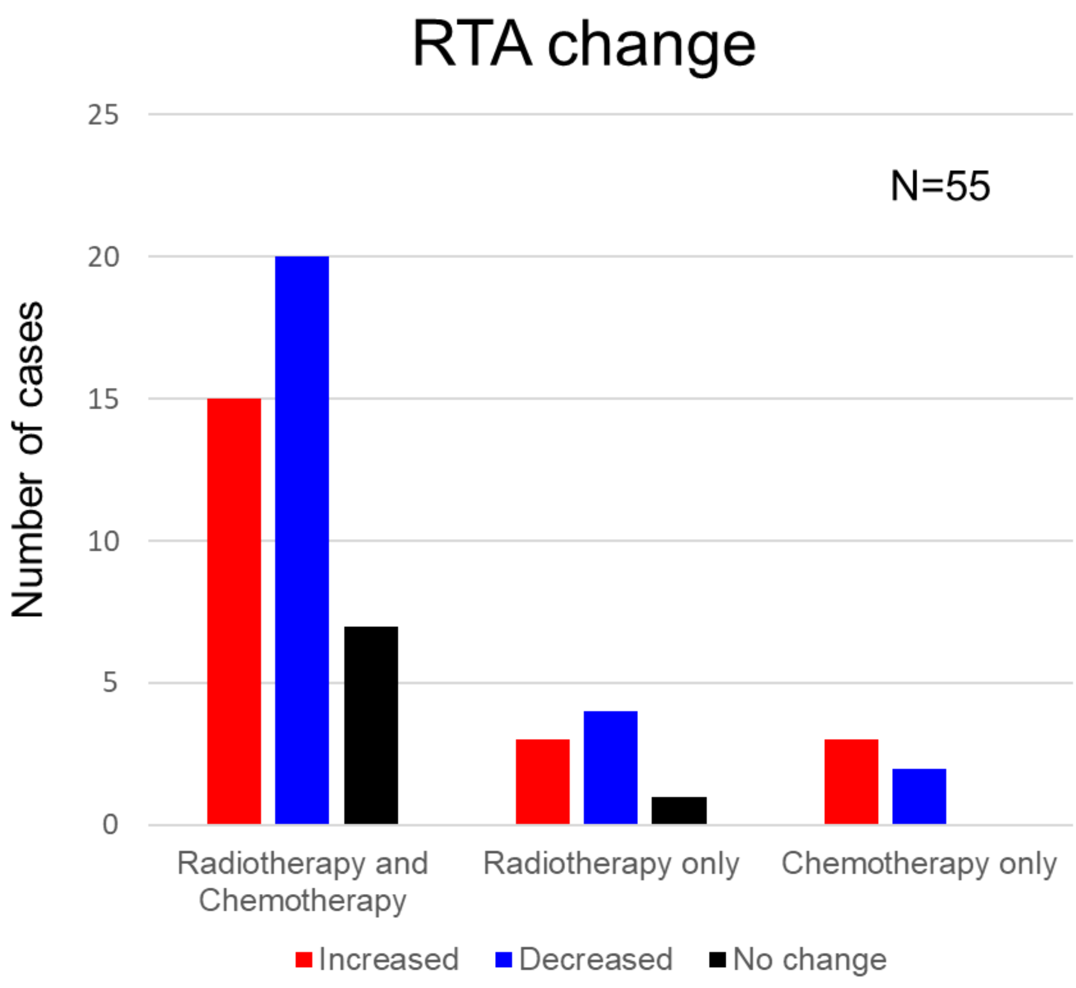

Supplement: Supplementary file 1 — Additional file 1: Figs. S1–S13. [file 13073_2022_1095_MOESM1_ESM.pdf]
